# Ketone Bodies Attenuate Wasting in Models of Atrophy

**DOI:** 10.1002/jcsm.12554

**Published:** 2020-04-02

**Authors:** Andrew P. Koutnik, Angela M. Poff, Nathan P. Ward, Janine M. DeBlasi, Maricel A. Soliven, Matthew A. Romero, Paul A. Roberson, Carl D. Fox, Michael D. Roberts, Dominic P. D'Agostino

**Affiliations:** ^1^ Department of Molecular Pharmacology and Physiology Morsani College of Medicine, University of South Florida Tampa FL USA; ^2^ Department of Cancer Physiology Moffitt Cancer Center, H. Lee Moffitt Cancer Center and Research Institute Tampa FL USA; ^3^ School of Kinesiology Auburn University Auburn AL USA; ^4^ Institute for Human and Machine Cognition Ocala FL USA

**Keywords:** Atrophy, Cachexia, Ketogenic, Ketones, Sepsis, Skeletal Muscle

## Abstract

**Background:**

Cancer Anorexia Cachexia Syndrome (CACS) is a distinct atrophy disease negatively influencing multiple aspects of clinical care and patient quality of life. Although it directly causes 20% of all cancer‐related deaths, there are currently no model systems that encompass the entire multifaceted syndrome, nor are there any effective therapeutic treatments.

**Methods:**

A novel model of systemic metastasis was evaluated for the comprehensive CACS (metastasis, skeletal muscle and adipose tissue wasting, inflammation, anorexia, anemia, elevated protein breakdown, hypoalbuminemia, and metabolic derangement) in both males and females. *Ex vivo* skeletal muscle analysis was utilized to determine ubiquitin proteasome degradation pathway activation. A novel ketone diester (*R/S* 1,3‐Butanediol Acetoacetate Diester) was assessed in multifaceted catabolic environments to determine anti‐atrophy efficacy.

**Results:**

Here, we show that the VM‐M3 mouse model of systemic metastasis demonstrates a novel, immunocompetent, logistically feasible, repeatable phenotype with progressive tumor growth, spontaneous metastatic spread, and the full multifaceted CACS with sex dimorphisms across tissue wasting. We also demonstrate that the ubiquitin proteasome degradation pathway was significantly upregulated in association with reduced insulin‐like growth factor‐1/insulin and increased FOXO3a activation, but not tumor necrosis factor‐α‐induced nuclear factor‐kappa B activation, driving skeletal muscle atrophy. Additionally, we show that *R/S* 1,3‐Butanediol Acetoacetate Diester administration shifted systemic metabolism, attenuated tumor burden indices, reduced atrophy/catabolism and mitigated comorbid symptoms in both CACS and cancer‐independent atrophy environments.

**Conclusions:**

Our findings suggest the ketone diester attenuates multifactorial CACS skeletal muscle atrophy and inflammation‐induced catabolism, demonstrating anti‐catabolic effects of ketone bodies in multifactorial atrophy.

## 1 Introduction

Cancer cachexia is a multifactorial atrophy syndrome characterized by skeletal muscle and often adipose tissue wasting that is typically accompanied by inflammation, anorexia, anemia, elevated protein breakdown, hypoalbuminemia, and metabolic derangement.^1^, ^2^, ^3^ Due to its multifaceted nature and complexity, cachexia is distinct from other forms of wasting and has been repeatedly shown to worsen prognosis, quality of life, and ability to receive, tolerate, and/or respond to standard of care for cancer patients. Consequently, 20% of cancer‐related deaths result from cachexia.^3^ Unfortunately, the prevalence of cachexia‐induced mortality in cancer patients remains unchanged from the initial mortality estimations in 1932,^4^, ^5^ as current modeling systems are limited and therapeutic interventions ineffective.^5^, ^6^, ^7^ While the terminology used to describe cancer cachexia has varied, cancer cachexia in the presence of comorbid anorexia is associated with higher morbidity and mortality, and is termed Cancer Anorexia Cachexia Syndrome (CACS).^8^

Defining the pathology of, and therapeutic response to, cachexia has largely relied on animal model systems^6^, ^7^; however, no established model comprehensively recapitulates the multifactorial CACS limiting clinical advancements. Of note, many model systems do not metastasize significantly despite consistent clinical reports demonstrating a higher incidence of cachexia and its comorbidities in the advanced metastatic stage.^7^, ^9^, ^10^, ^11^ Furthermore, emergent evidence indicates that the metastatic process may contribute to cachexia onset and progression.^12^ In fact, it has been recently argued that the inability to model metastasis may be contributing to reduced bench to bed side translation.^7^ The VM‐M3 mouse model of systemic metastasis (VM‐M3) is a novel syngeneic mouse model derived from a spontaneous glioma tumor which, when implanted subcutaneously, reliably recreates the spontaneous metastatic process (primary tumor formation, followed by establishment of distal secondary tumors), resulting in systemic metastasis to the liver, lungs, brain, kidneys, and spleen.^13^, ^14^ Additionally, the model is luciferase tagged, permitting *in vivo* bioluminescent‐based monitoring of disease progression. This convenient and consistent model system exhibits many of the hallmarks of the metastatic condition, mirroring important features of clinical cachexia and addressing the overt gap in current cachexia modeling systems, yet until now, it has not been analyzed to determine if it exhibits the comprehensive CACS.

While current models are a critical limitation to clinical advancement, a lack of effective and/or supportive treatments remains a century old problem.^4^, ^5^ However, work dating back a half‐century illustrated that patients who underwent extreme nutrient deprivation produced endogenous metabolites called ketone bodies that accompanied altered systemic metabolism and skeletal muscle protein kinetics which was hypothesized to prolong life during catabolic states by attenuating muscle atrophy.^15^ Additionally, dietary or infusion‐induced elevations of ketone bodies have been linked to altered metrics of protein breakdown across various atrophy and post‐absorptive conditions.^16^, ^17^, ^18^, ^19^, ^20^, ^21^, ^22^ These ketone bodies, which are the end‐product of fatty acid oxidation and subsequent ketogenesis in the hepatic tissue, are shown to be upregulated during fasting, reduced carbohydrate metabolism, and/or direct intravenous (IV) infusion. Yet, dietary restriction may be contraindicated in atrophy‐based disease/environments, and IV infusion is limited to the clinical/research environment. Recent development of orally administrated exogenous ketone molecules is circumventing previous barriers to ketone body elevation and has led to the rapid expansion of research unveiling direct metabolite effects on metabolism, inflammation, oxidative stress, and epigenetic regulation, among others.^23^, ^24^, ^25^, ^26^ While our team hypothesized that exogenous ketone bodies may mitigate inflammatory‐based atrophy disease,^27^ their chronic anti‐catabolic effects in multifactorial wasting diseases have remained untested.

The purpose of this study was twofold: (a) to evaluate the VM‐M3 model for the full multifactorial CACS as it is currently presented in the clinical environment and (b) to determine if exogenous oral administration of ketone bodies could attenuate atrophy in both the full progressive metastatic CACS and in a cancer‐independent inflammation atrophy environment when controlling for confounding and comorbid variables. We hypothesized that the VM‐M3 model would more accurately recapitulate the clinical diagnostic hallmarks of CACS and that exogenous administration of ketone bodies would attenuate atrophy in both the cancer and cancer‐independent atrophy environments. Our findings suggest that the VM‐M3 model presents a novel, immunocompetent, logistically feasible, and repeatable phenotype with progressive tumor growth, spontaneous metastatic spread, and the full CACS with sex dimorphisms across tissue wasting. This model addresses three current gaps in CACS modeling: (a) the progressive spontaneous metastatic environment, (b) the full clinical syndrome of progressive wasting of skeletal muscle with altered insulin‐like growth factor‐1 (IGF‐1)/insulin ubiquitin proteasome degradation pathway, adipose tissue wasting, systemic inflammation, anorexia, anemia, hypoalbuminemia, elevated protein breakdown, and metabolic derangement, and (c) sex‐specific temporal cachectic discrepancies. Additionally, our work demonstrates that oral administration of *R/S* 1,3‐Butanediol Acetoacetate Diester, a novel synthetic ketone diester (KDE), supplemented in addition to a standard diet, was well‐tolerated, diminished anorexia, induced alterations in systemic metabolism, attenuated tumor burden indices, and reduced skeletal muscle atrophy despite circulating anabolic hormones, IGF‐1 and insulin, remaining significantly reduced. The current work also demonstrates that the KDE mitigated catabolism in a cancer‐independent multifactorial inflammation‐induced atrophy environment of lipopolysaccharide (LPS)‐induced sepsis, even when controlling for confounding and comorbid variables. Taken together, the VM‐M3 model has the full progressive multifactorial CACS, and KDE attenuates multifactorial CACS and inflammatory atrophy.

## 2 Methods

### 2.1 Cell Culture

VM‐M3 cells were derived from a spontaneous tumor in a VM/Dk inbred mouse and adapted to cell culture.^13^ VM‐M3 cells were transduced with a lentivirus vector containing the firefly *luciferase* gene under control of the cytomegalovirus promoter as previously described.^13^ VM‐M3 cells were cultured in d‐glucose, l‐glutamine, and sodium pyruvate‐free Dulbecco's modified Eagle medium (Gibco, Life Technologies) supplemented with 10% fetal bovine serum (Invitrogen), 25‐mM d‐glucose (Fisher Scientific), 2‐mM l‐glutamine (Gibco, Life Technologies), 1% penicillin‐streptomycin (Invitrogen), and 10‐mM HEPES buffer (Gibco, Life Technologies). Cells were maintained at 37 °C in 95% air, 5% CO_2_ in a humidified incubator.

### 2.2 Animals

Five breeding pairs of the VM/Dk strain of mice were used to establish and propagate a VM/Dk mouse colony in the University of South Florida (USF) Morsani College of Medicine Vivarium according to standard husbandry protocol. Male and female VM/Dk mice between 10 and 23 weeks of age were singly housed to accurately assess individual animal food intake. Bodyweight and food intake were tracked daily prior to and after social isolation to ensure bodyweight and food intake normalized prior to cell inoculation (*Table* S1, supporting information). Animals were distributed into one of four groups: sham male (SH‐M), sham female (SH‐F), cancer male (CA‐M), and cancer female (CA‐F). Each cancer animal was matched with a sham animal of equivalent sex, bodyweight, age, and food intake (*Figures* S1A and S1B) to ensure appropriate cancer and sex‐specific comparisons. To determine KDE‐induced effects in CACS, VM/Dk animals were distributed into one of three groups: KDE + VM‐M3, VM‐M3, and Sham with equivalent sex, bodyweight, age and food intake at baseline (*Figure* S6A). One unexplained premature death occurred in SH‐F (Experiment 1a) and Sham (Experiment 4c) groups immediately post‐inoculation. To determine KDE‐induced effects post‐LPS administration, VM/Dk animals were distributed into two groups (*Figure* S7A and S7B): KDE + LPS and LPS of equivalent bodyweights. All procedures were approved by the USF Institutional Animal Care and Use Committee (Protocol Numbers R1900 & R5829) and performed under strict adherence to the NIH Guide for the Care and Use of Laboratory Animals.

### 2.3 VM‐M3 Cell and LPS Implantation

**Figure 1:**
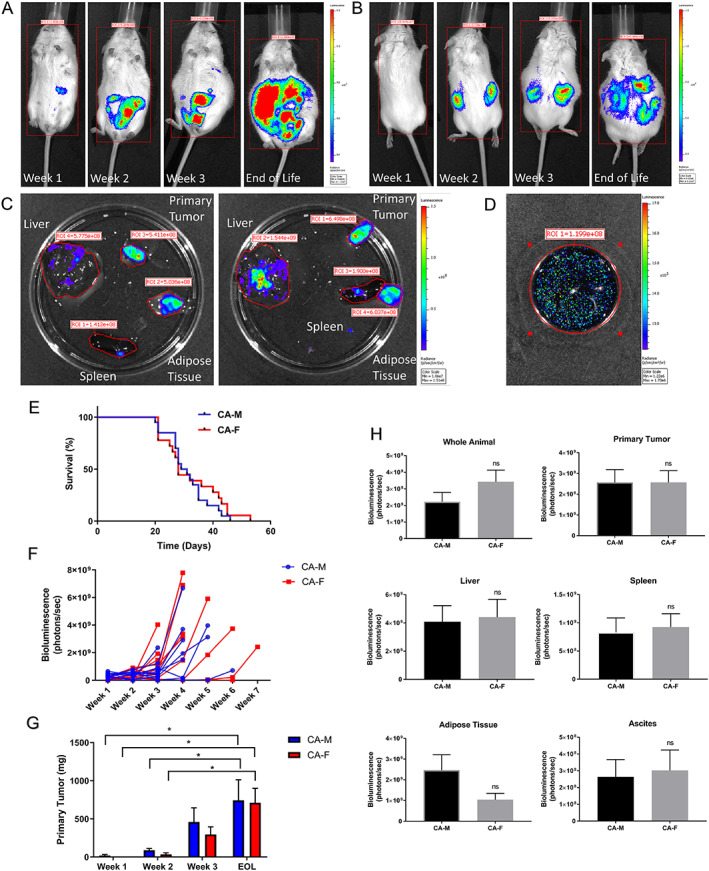
**VM‐M3 Presents with Progressive Tumor Growth and Spontaneous Systemic Metastases**. **(A,B)** Weekly *in vivo* IVIS luciferin imaging for representative animal cancer‐female #3 (CA‐F3) in the prone and supine positions, respectively. Primary tumor is visible Week 1, subsequent progressive metastases starting Week 2 until end of life (EOL). Red line indicates region of interest (ROI). Color scale/radiance: 3.62e6–1.21e7 photons/s/cm^2^/sr. Data: Experiment 1a. **(C)** *Ex vivo* anterior and posterior view of CA‐F3's organ tissues (liver, spleen, adipose tissue, and primary tumor) via IVIS luciferin imaging indicates metastatic spread. Red line indicates region of interest (ROI). Color scale/radiance: 1.06e7–1.51e8 photons/s/cm^2^/sr. Data: EOL, Experiment 1a. **(D)** *Ex vivo* ascites fluid IVIS luciferin imaging shows presence of VM‐M3 cells. Red line indicates ROI. Color scale/radiance: 1.22e6–1.70e6 photons/s/cm^2^/sr. Data: EOL, Experiment 1a. **(E)** Survival curve for cancer males (CA‐M, *n* = 20) and females (CA‐F, *n* = 18) shows no significant differences. Data: Experiment 1a and b. **(F)** CA‐M (*n* = 12) and CA‐F (*n* = 12) data points represent the sum of both prone and supine *in vivo* bioluminescence for each individual animal. Tumor burden was progressive. Data: Experiment 1a. **(G)** Primary tumor weight progressively increased in both CA‐M (Week 1, *n* = 4; Week 2, *n* = 4; Week 3, *n* = 5; EOL, *n* = 11) and CA‐F (Week 1, *n* = 4; Week 2, *n* = 5; Week 3, *n* = 6; EOL, *n* = 10) Week 1 to EOL. Primary tumor weight at EOL was significantly larger than Weeks 2 and 3 for both CA‐M and CA‐F. Data: Weeks 1–3, Experiment 2; EOL, Experiment 1a. **(H)** Whole animal *in vivo* (*n* = 12/group) and *ex vivo* IVIS luciferin imaging (Primary Tumor, CA‐M *n* = 11, CA‐F *n* = 9; liver, spleen, CA‐M *n* = 12, CA‐F *n* = 12; intraperitoneal adipose tissue, CA‐M *n* = 12, CA‐F *n* = 10; ascites fluid, CA‐M *n* = 7, CA‐F *n* = 5) indicate similar tumor burden. Data: EOL, whole animal, primary tumor, liver, spleen, and intraperitoneal adipose tissue, Experiment 1a; ascites fluid, Experiment 1b. **Data Information:** Kaplan‐Meier analysis and log‐rank test (E). Within group differences across time were analyzed with one‐way ANOVA with Tukey's post hoc (G). Differences across groups were analyzed with unpaired *t* test (G,H). Colors (E‐G): CA‐M, blue; CA‐F, red. Data are mean ± *SEM*. ^**ns**^ ***P*** > 0.05, * ***P*** < 0.05.

**Figure 2:**
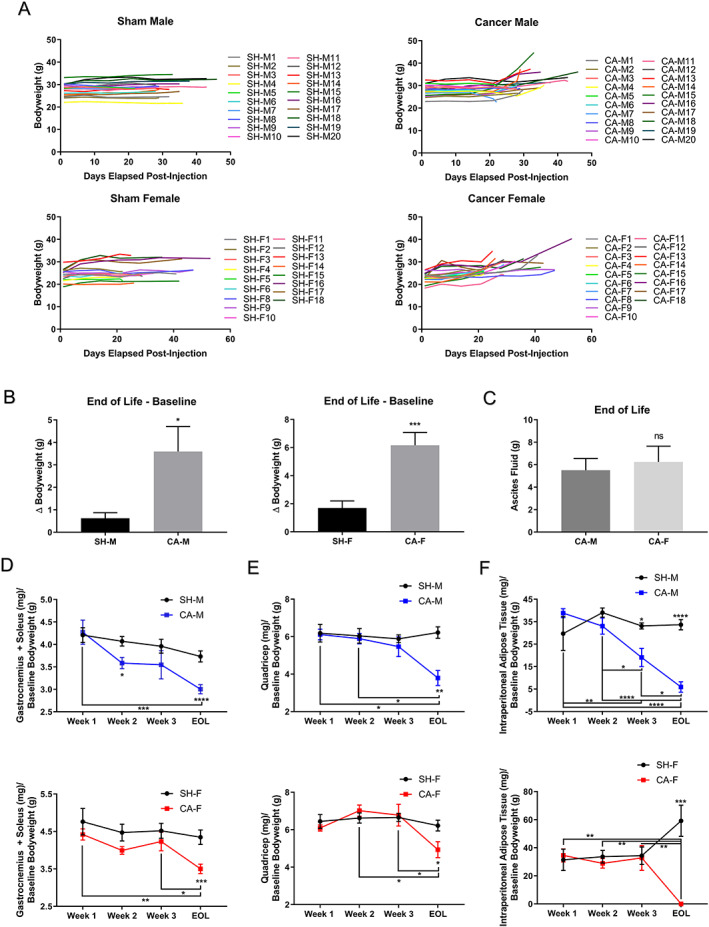
**VM‐M3 Develop Skeletal Muscle and Adipose Tissue Wasting Not Represented in Bodyweight Measurements**. (**A**) Sham males (SH‐M, *n* = 20), cancer males (CA‐M, *n* = 20), sham females (SH‐F, *n* = 17), and cancer females (CA‐F, *n* = 18) bodyweight. Data: Experiment 1a and b. (**B**) Change in bodyweight across groups (SH‐M, *n* = 20), (CA‐M, *n* = 20), (SH‐F, *n* = 17), and (CA‐F, *n* = 18). Data: Experiment 1a and b. (**C**) Ascites fluid weight in CA‐M (5.5 g, *n* = 8) and CA‐F (6.2 g, *n* = 6). Data: EOL, Experiment 1b. (**D**) Gastrocnemius and soleus weights as a ratio to baseline bodyweight for SH‐M, CA‐M (M, Week 1, *n* = 4; Week 2, *n* = 4; Week 3, *n* = 5; EOL, *n* = 20), SH‐F, CA‐F (F, Week 1, *n* = 5; Week 2, *n* = 5; Week 3, *n* = 6; EOL, *n* = 17). Data: Weeks 1–3, Experiment 2; EOL, Experiment 1a and b. (**E**) Quadricep weights as a ratio to baseline bodyweight for SH‐M, CA‐M (M, Week 1, *n* = 4; Week 2, *n* = 4; Week 3, *n* = 5; EOL, *n* = 3), SH‐F, CA‐F (F, Week 1, *n* = 5; Week 2, *n* = 5; Week 3, *n* = 6; EOL, *n* = 4). Data: Weeks 1‐3, Experiment 2; EOL, Experiment 1b. (**F**) Intraperitoneal adipose tissue weights as a ratio to baseline bodyweight tissue weights for SH‐M, CA‐M (M, Week 1, *n* = 4; Week 2, *n* = 4; Week 3, *n* = 5; EOL, *n* = 8), SH‐F, CA‐F (F, Week 1, *n* = 5; Week 2, *n* = 5; Week 3, *n* = 6; EOL, *n* = 6). Data: Week 1‐3, Experiment 2; EOL, Experiment 1b. **Data Information**: Within group differences across time were analyzed with one‐way ANOVA with Tukey's post hoc (D‐F). Differences across groups at each time point were analyzed with unpaired *t* test (B‐F). Colors (D‐F): CA‐M, blue; CA‐F, red. Data are mean ± *SEM*. ^**ns**^ ***P*** > 0.05, * ***P*** < 0.05, ** ***P*** < 0.01, *** ***P*** < 0.001, **** ***P*** < 0.0001.

**Figure 3:**
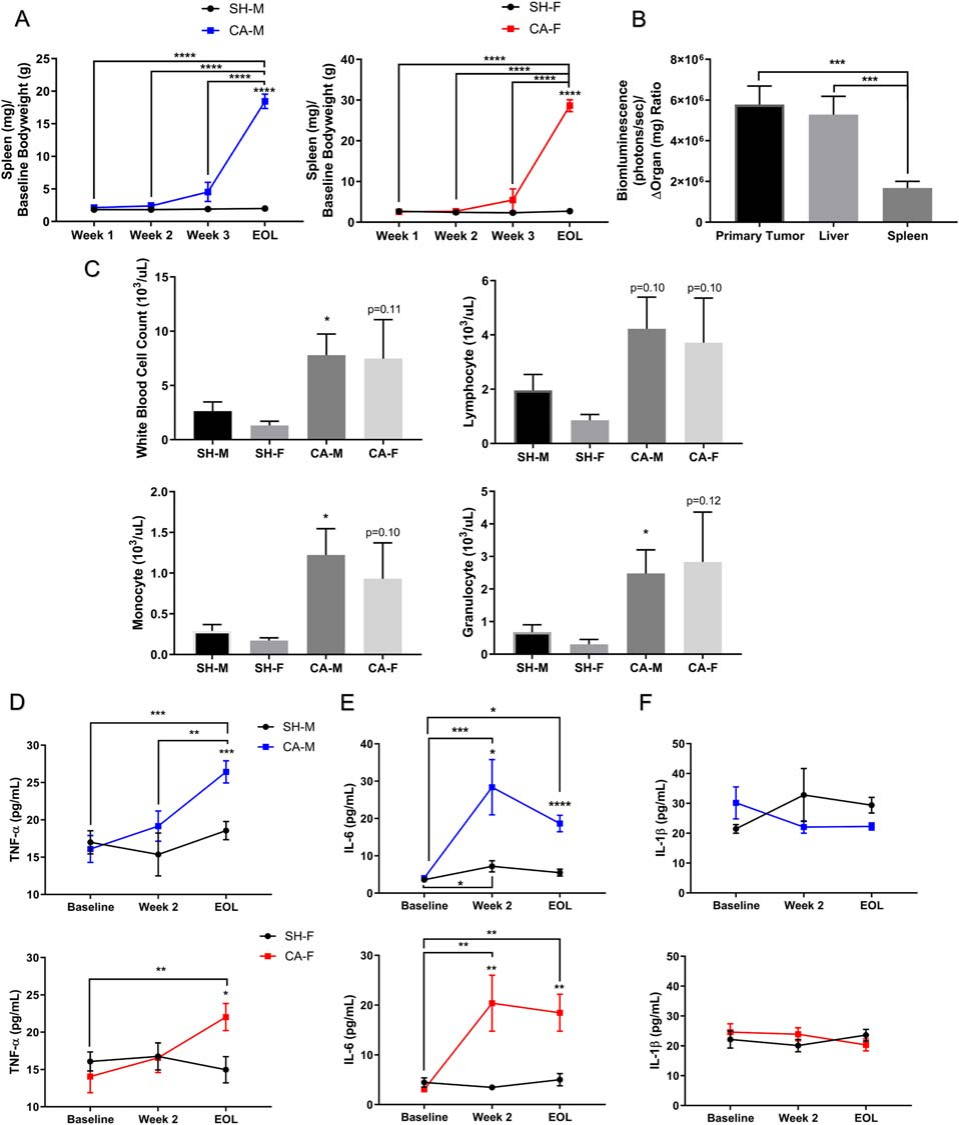
**VM‐M3 Develop Prolonged Systemic Inflammation**. (**A**) Spleen weights as a ratio to baseline bodyweight for sham males (SH‐M), cancer males (CA‐M) [M, Week 1, *n* = 4; Week 2, *n* = 4; Week 3, *n* = 5; end of life (EOL), *n* = 20], sham females (SH‐F), and cancer females (CA‐F) (F, Week 1, *n* = 5; Week 2, *n* = 5; Week 3, *n* = 6; EOL, *n* = 17). Data: Weeks 1–3, Experiment 2; EOL, Experiment, 1a and b. (**B**) Primary tumor (*n* = 20), liver (*n* = 24), and spleen (*n* = 24) weight change to bioluminescence ratio via IVIS luciferin imaging. Data: EOL, Experiment 1a. (**C**) White blood cell count analysis via impedance analysis (SH‐M, *n* = 9; SH‐F, *n* = 10; CA‐M, *n* = 9; CA‐F, *n* = 10). Data: EOL, Experiment 1a and b. (**D‐F**) Cytokines quantification via Luminex fluorophore intensity analysis (*n* = 9–12/group/time point). Data: Experiment 1a. **Data Information**: Within and across group differences were analyzed with one‐way ANOVA with Tukey's post hoc with >3 comparisons (A) and Fischer LSD post‐hoc for ≤3 comparisons (B,D‐F). Differences across groups (A,D‐F) or within sexes (C) at each time point were analyzed with unpaired *t* test. Prior to ANOVA cytokine analysis, robust regression and outlier removal (ROUT) with coefficient *Q* = 1% was used as non‐physiologic/error values were detected (D‐F). Colors (A,D‐F): CA‐M, blue; CA‐F, red. Data are mean ± *SEM*. * ***P*** < 0.05, ** ***P*** < 0.01, *** ***P*** < 0.001, **** ***P*** < 0.0001.

**Figure 4:**
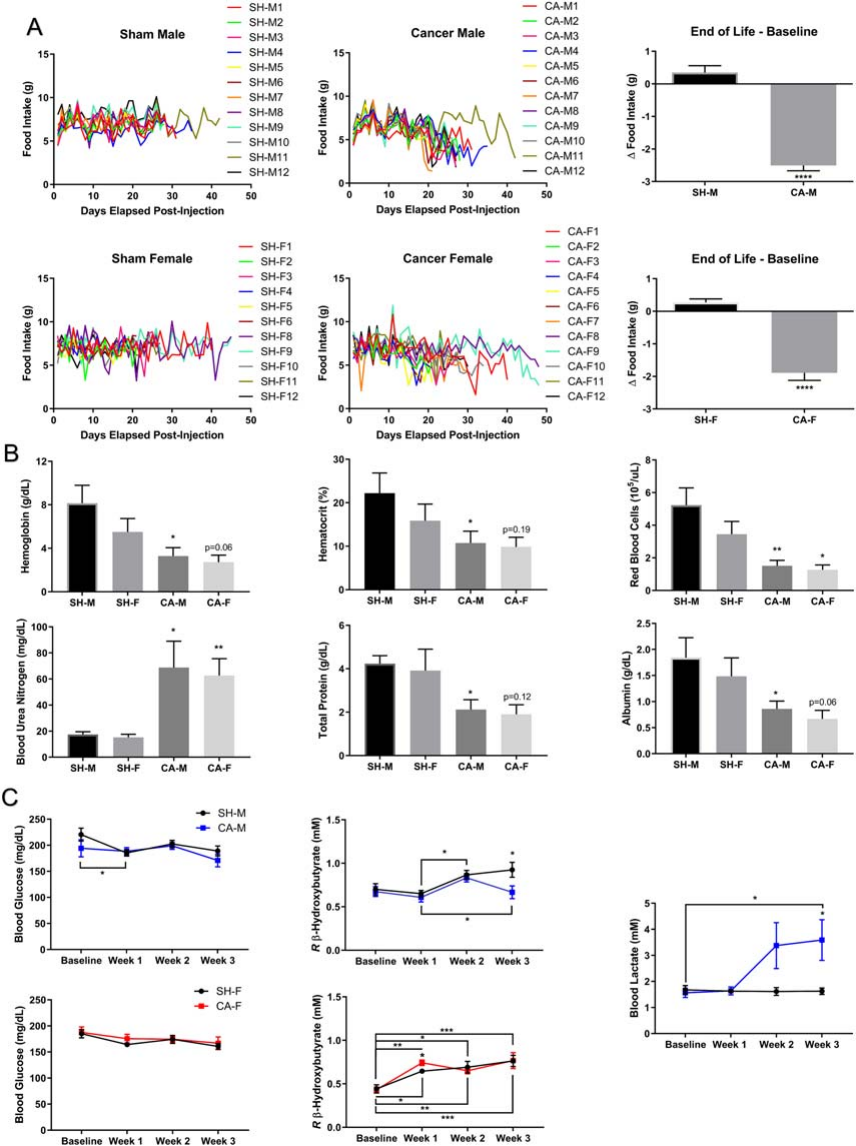
**VM‐M3 Develop Anorexia, Anemia, Protein Breakdown, Hypoalbuminemia, and Metabolic Derangement**. **(A)** Food Intake was tracked with 4‐day average analyzed at end of life (EOL) to baseline (*n* = 12/group). Data: Experiment 1. (**B**) Hemoglobin (*n* = 10/group), Hematocrit (males *n* = 10/group; females *n* = 9/group), and red blood cell count (*n* = 10/group) were quantified with impedance analysis. Blood urea nitrogen (*n* = 5/group), total protein (males *n* = 9/group; females *n* = 5–6/group), and albumin (males *n* = 5/group; females *n* = 6/group) were analyzed via colorimetry analysis. Data: EOL, Experiment 1a and b. (**C**) Blood glucose, ketones, and lactate were quantified via enzyme interaction (*n* = 8–12/group). Data: blood glucose and ketones, Experiment 1a; blood lactate, Experiment 3. **Data Information**: Within group differences across time were analyzed with one‐way ANOVA with Tukey's post hoc with >3 comparisons (C). Differences across groups (A,C) or within sexes (B) at each time point were analyzed with unpaired *t* test. Abbreviations: SH‐M, sham males; CA‐M, cancer males; SH‐F, sham females; CA‐F, cancer females. Colors (C): CA‐M, blue; CA‐F, red. Data are mean ± *SEM*. * ***P*** < 0.05, ** ***P*** < 0.01, *** ***P*** < 0.001, **** ***P*** < 0.0001.

**Figure 5:**
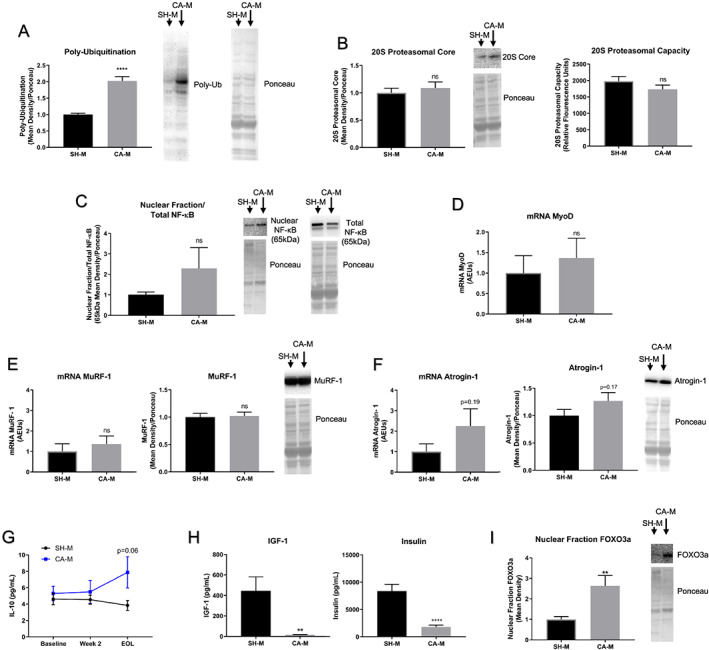
**IGF‐1/Insulin‐FOXO3a‐Ubiquitin Proteasome Pathway is Activated in VM‐M3 Skeletal Muscle**. (**A**) Immunoblotting and quantitative densitometry analysis indicated poly‐ubiquitination was significantly increased in CA‐M compared to SH‐M (*n* = 12). Data: Gastrocnemius, EOL, Experiment 1a. (**B**) Immunoblotting and quantitative densitometry analysis indicated 20S proteasome core was not changed in CA‐M compared to SH‐M (*n* = 12/group). Fluorometric enzymatic assay indicated no change in 20S proteasome capacity (*n* = 12/group). Data: Gastrocnemius, EOL, Experiment 1a. (**C**) Immunoblotting total and nuclear fraction NF‐κB (65 kDa) and ponceau staining. Densitometry quantification did not indicate altered NF‐κB activation between SH‐M (*n* = 11) and CA‐M (*n* = 12). Data: Gastrocnemius, EOL, Experiment 1a. (**D**) RT‐qPCR analysis of mRNA MyoD in SH‐M and CA‐M revealed no differences (*n* = 12/group). Data: Gastrocnemius, EOL, Experiment 1a. (**E**) RT‐qPCR analysis of mRNA MuRF‐1, immunoblotting and quantitative densitometry analysis of MuRF‐1 in SH‐M and CA‐M revealed no differences (*n* = 12/group). Data: Gastrocnemius, EOL, Experiment 1a. (**F**) RT‐qPCR analysis of mRNA Atrogin‐1, immunoblotting and quantitative densitometry analysis of Atrogin‐1 in SH‐M and CA‐M revealed non‐significant elevations (*n* = 12/group). Data: Gastrocnemius, EOL, Experiment 1a. (**G**) Cytokines quantification via Luminex fluorophore intensity analysis (*n* = 7–9/group) indicate elevations in IL‐10. Data: Experiment 1a. (**H**) Serum hormone levels quantified via Luminex xMAP fluorophore intensity analysis demonstrated 26‐fold reduction in insulin‐like growth factor‐1 (IGF‐1) and 4.5‐fold reduction in Insulin in CA‐M compared to SH‐M (*n* = 11/group). Data: Week 3, Experiment 4c. (**I**) Immunoblotting and quantitative densitometry analysis indicated nuclear fraction FOXO3a was significantly increased (2.4‐fold) in CA‐M (*n* = 12) compared to SH‐M (*n* = 11). Data: Gastrocnemius, EOL, Experiment 1a. **Data Information**: Within group differences across time were analyzed with one‐way ANOVA with Fischer LSD post hoc for ≤3 comparisons (G). Differences across groups at each time point were analyzed with unpaired *t* test (A‐I). Prior to ANOVA cytokine analysis, robust regression and outlier removal (ROUT) with coefficient *Q* = 1% was used as non‐physiologic/error values were detected (G). Colors (G): CA‐M, blue. Data are mean ± *SEM*. ^**ns**^ ***P*** > 0.05, ** ***P*** < 0.01, **** ***P*** < 0.0001.

**Figure 6:**
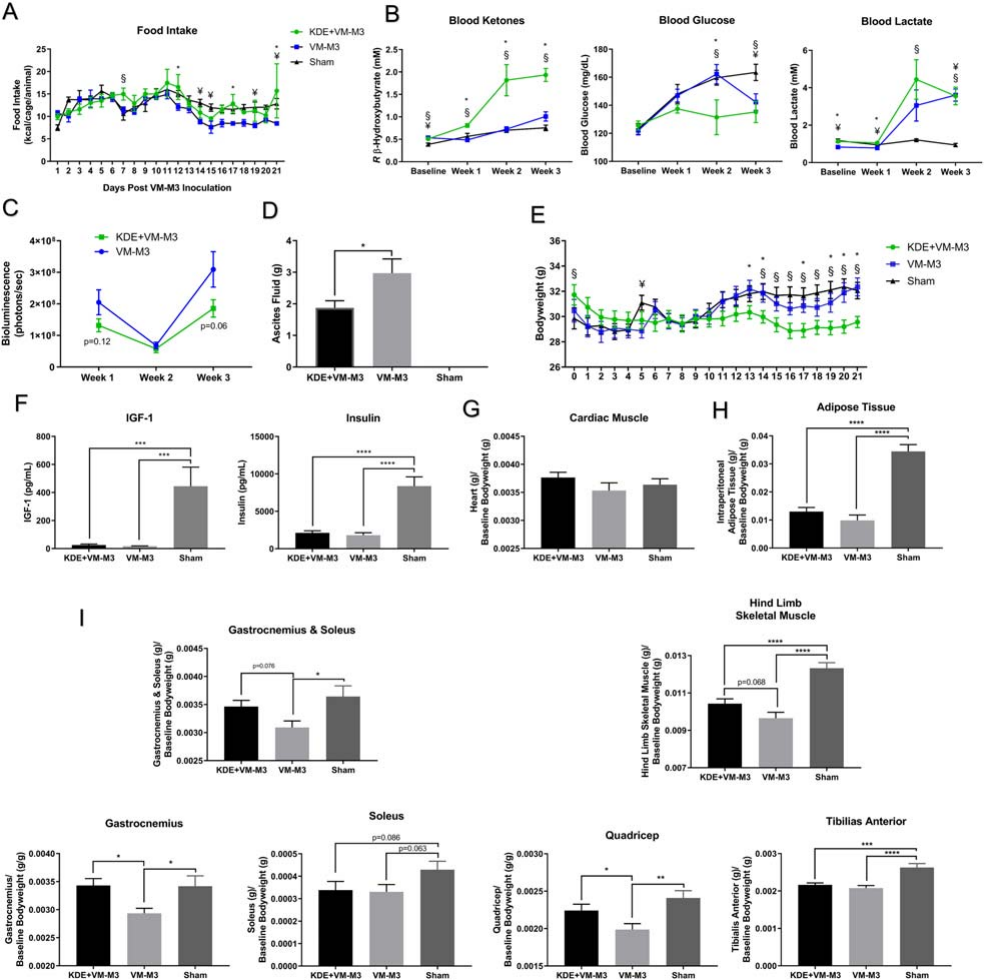
**Ketone Diester Mitigates Comorbidities, Tumor Burden Indices, and Skeletal Muscle Atrophy in Cancer Anorexia Cachexia Syndrome**. (**A**) Food intake was tracked daily and compared across groups. (*n* = 3/group; group = cage; 3‐4 animals/cage). Data: Experiment 4c. (**B**) Blood ketones, glucose, and lactate were quantified for Ketone Diester + VM‐M3 cancer anorexia cachexia syndrome (KDE + VM‐M3, *n* = 12), VM‐M3 (*n* = 12), and sham (*n* = 11) via enzyme interaction. KDE + VM‐M3 demonstrated shifts in system metabolism. Data: Experiment 4c. (**C**) KDE + VM‐M3 (*n* = 12) and VM‐M3 (*n* = 12) data points represent the average of both prone and supine *in vivo* bioluminescence for each individual animal. KDE + VM‐M3 has non‐significant reductions in tumor burden. Data: Experiment 4c. (**D**) Ascites fluid weight in KDE + VM‐M3 (1.9 g, *n* = 11), VM‐M3 (3.0 g, *n* = 11), and sham (0 g, *n* = 11). Data: Week 3, Experiment 4c. (**E**) Daily bodyweight for KDE + VM‐M3 (*n* = 12), VM‐M3 (*n* = 12), and Sham (*n* = 11). VM‐M3 and sham saw an increase in bodyweight compared to KDE‐VM‐M3, starting Day 14. Data: Experiment 4c. (**F**) Serum hormone levels quantified via Luminex fluorophore intensity analysis demonstrated significant reductions in insulin‐like growth factor‐1 (IGF‐1) and insulin in KDE‐VM‐M3 (*n* = 12) and VM‐M3 (*n* = 11) compared to SH‐M (*n* = 11). Data: Week 3, Experiment 4c. (**G**) Cardiac tissue weight set to baseline bodyweight (*n* = 10/group) with no significant difference across groups. Data: Week 3, Experiment 4c. (**H**) Intraperitoneal adipose tissue weight set to baseline bodyweight with significant reductions in KDE + VM‐M3 and VM‐M3 compared sham (*n* = 10/group). Data: Week 3, Experiment 4c. (**I**) Gastrocnemius, soleus, quadricep, tibialis anterior, and hind limb (four muscle pooled analysis) skeletal muscle weights set to baseline bodyweight showed KDE + VM‐M3 attenuated skeletal muscle atrophy across several tissues compared to VM‐M3 (*n* = 10/group). Data: Week 3, Experiment 4c. **Data Information**: Differences across groups at each time point were analyzed with one‐way ANOVA with Fischer LSD post hoc for ≤3 comparisons (A,B,E‐I) or unpaired *t* test for individual comparisons (C,D). Colors (A‐C,E): KDE + VM‐M3, green; VM‐M3, blue. Data are mean ± *SEM*. KDE‐VM‐M3 versus VM‐M3 * ***P*** < 0.05, KDE‐VM‐M3 versus sham ^**§**^ ***P*** < 0.05, VM‐M3 versus sham ^**¥**^ ***P*** < 0.05 (A,B,E). ^**ns**^ ***P*** > 0.05, * ***P*** < 0.05, ** ***P*** < 0.01, *** ***P*** < 0.001, **** ***P*** < 0.0001 (C,D,F‐I).

For VM‐M3 implantation in the model characterization experiments (*Figures* 1, 2, 3, 4, 5; *Figures* S1–S5), ~1 × 10^6^ VM‐M3 cells (T. Seyfried, Boston College) in 300‐μl sterile PBS (CA‐M and CA‐F) or 300‐μl sterile PBS only (SH‐M and SH‐F) were injected subcutaneously into the left abdominal flank resulting in primary tumor formation at the injection site and subsequently systemically metastasizes to most major organs, namely, the liver, kidneys, spleen, lungs, and brain as previously described.^13^, ^14^ Additional model analysis revealed metastatic disease and CACS temporal progression could be replicated with intraperitoneal administration of VM‐M3 cells (*Table* S2). This method of implantation further minimized variability in markers of tumor progression and therefore provides a useful optional method technique in the VM‐M3 model for efficient analysis of potential therapeutic agents. To determine KDE‐induced effects on CACS, 1 × 10^6^ VM‐M3 cells in 300‐μl sterile PBS (KDE + VM‐M3 and VM‐M3) or 300‐μl sterile PBS only (Sham) were administered intraperitoneally (*Figure* 6; *Figure* S6). For LPS administration in the septic atrophy model, LPS (Escherichia coli O55:B5; L2880; Sigma‐Aldrich, St. Louis, MO, USA) was diluted in sterile PBS and administered intraperitoneally at 10 mg/kg as pilot work indicated a maximal, yet nonfatal, ~15% bodyweight reduction at 10 mg/kg dose.

### 2.4 Survival Analysis

Animal health and behavior were assessed daily. VM‐M3 animals were humanely euthanized together with their sham‐matched controls (sex, bodyweight, age, and food intake matched at baseline) by exsanguination under isoflurane (Henry Schein Animal Health, Dublin, OH, USA) according to Institutional Animal Care and Use Committee guidelines upon presentation of end of life (EOL) defined criteria associated with tumor burden and disease progression (decreased response to stimuli, failure to thrive, labored breathing and/or locomotion, and/or complete cessation of food intake). Survival time was recorded.

### 2.5 Tumor Growth and Metastasis

Tumor growth was monitored weekly as a measure of bioluminescence using the Xenogen IVIS Spectrum system (Caliper LS, Hopkinton, MA, USA). Data acquisition and analysis was performed using the Living Image® software (Caliper LS). Approximately 15 min prior to *in vivo* imaging, VM/Dk mice received an intraperitoneal injection of d‐Luciferin (50 mg/kg; 88293, Thermo Fisher Scientific; Waltham, MA, USA). Bioluminescent signal was obtained using the IVIS cooled charge‐coupled device (CCD) camera system in both prone and supine positions. As only the cancer cells were transfected with the luciferase gene, bioluminescent signal (photons/s) of the whole animal was measured and tracked over time as an indicator of tumor burden and metastatic spread. At EOL, primary tumor, spleen, liver, and adipose tissue were gathered and saturated with d‐Luciferin (10‐μl d‐Luciferin + PBS/g tissue at 5 mg d‐Luciferin/ml PBS dilution) for 5 min and imaged to determine tumor burden. Ascites fluid was imaged 15 min after resuspension with d‐Luciferin (20‐μl d‐Luciferin+PBS/ml ascites fluid at 5 mg d‐Luciferin/ml PBS dilution) to assess presence of circulating tumor cells.

### 2.6 Body Composition

Bodyweight was assessed daily at the same time (7:00–9:00 a.m.). At EOL, bodyweight and weights of ascites fluid, primary tumor, calf (combined gastrocnemius & soleus), anterior thigh (quadriceps), intraperitoneal fat pads, liver, and spleen tissue were measured (*Table* S1; Experiment 1a & b). A follow‐up time, course experiment was conducted at Weeks 1, 2, and 3 to assess weekly changes in bodyweight and aforementioned tissues for both cancer and sham‐matched control animals (Experiment 2). For follow up evaluation of KDE in CACS, bodyweight was tracked daily with tissue weight determined at 21 days, prior to EOL (Experiment 4c). All tissue weights were gathered at harvest and normalized to baseline bodyweight (not influenced by cachexia progression) to allow for appropriate comparison between animals. For evaluation of KDE in LPS, bodyweight was tracked daily for evaluation pre‐ and post‐LPS administration (Experiment 5b & c).

### 2.7 Food Intake and Ketone Diester Administration

Standard diet dry food (2018 Teklad Global 18% protein rodent diet, Harlan) was mixed with deionized water (1 g dry food/ml deionized water) into a consistent paste and placed on a 100 × 15‐mm dish. Food intake was tracked daily at the same time (7:00–9:00 a.m.) and replaced every other day to ensure fresh food. Due to sinusoidal/oscillatory changes in food intake observed every other day (2‐day pattern), a 4‐day (2 × 2‐day pattern) average was taken at EOL and baseline to calculate changes in anorexic symptoms. KDE was chemically synthesized with physical properties, preparation, and analysis described in *Tables* S3–S4. For evaluation of KDE effect on anorexia, food intake was tracked daily at the same time (7:00–9:00 a.m.). As pilot work indicated reductions in ad libitum food intake with 20–30% by weight KDE incorporation standard diet, 1%/weight saccharine (Sigma‐Aldrich) and 5%/weight peanut butter (Natural Jif Creamy, J.M. Smucker Company, Orrville, OH, USA) were added to paste to increase palatability of standard diet across groups (Highly Palatable Standard Diet; HPSD). Additional pilot work revealed that incremental incorporation of KDE from 0% to 30% KDE/weight at 5% KDE/weight/day was better tolerated and did not result in changes in bodyweight across time (*Figure* S6B). Consequently, upon VM‐M3 inoculation, KDE + VM‐M3 received ad libitum 0% KDE/weight Day 1, 5% KDE Day 2, 10% KDE Day 3, 15% KDE Day 4, 20% KDE Day 5, 25% KDE Day 6, and 30% KDE Day 7 through 21 on top of HPSD. VM‐M3 and sham received HPSD ad libitum. As pilot work indicated potential water evaporation in food paste in ventilated cages, dehydration standard curve was calculated across various volumes of plated food (5, 10, 25, 50, 100 g per food) with and without KDE over a 2‐day period. Standard curve equation [water evaporated = 0.2472 (original grams of water) + 1.364] was used to correct for amount of dehydration prior to calculating caloric intake. For LPS experiments, 1 × 4 ml/kg KDE gavage or 1 × 4 ml/kg water gavage were administered for KDE + LPS and LPS only, respectively. Ab libitum food intake was tracked daily during initial analysis with caloric restriction reported as the percent reduction in 24‐hr caloric intake from pre‐ to post‐LPS administration. Pair feeding was conducted during subsequent analysis. Pair feeding was accomplished by measuring the 24‐hr caloric intake of LPS‐only group and presenting this amount of food to the KDE + LPS group.

### 2.8 Inflammation

Whole blood was gathered at baseline, Week 2, and EOL prior to anesthetics exposure via submandibular puncture to prevent anesthetic influence on inflammatory biomarkers^28^ and to avoid potential ascites contamination from cardiac puncture. A 60 μl of whole blood was placed into a K_2_EDTA tube (BD Microtainer, Franklin, NJ, USA), relabeled for blinded analysis, and analyzed via HemoTrue (Heska, Loveland, CO, USA) to assess white blood cell counts. Remaining whole blood was placed into serum separator tubes (MiniCollect 0.8 ml, Kremsunster, Austria) and centrifuged (13,000 rpm, 4 **°**C, 15 min) to isolate serum. A 25 μl of serum was mixed with 25 μl of saline, relabeled for blinded analysis and analyzed using Bio‐Plex (Bio‐Rad, Hercules, CA, USA) fluorescent bead technology to generate cytokine concentrations via a standard curve (EVE Technology Mouse Cytokine/Chemokine Array 31‐Plex). To determine whether spleen or liver enlargement in VM‐M3 animals was explained by tumor burden alone or other immunologic factors, tissue bioluminescence was divided by the difference in tissue weight between VM‐M3 and sham‐matched controls. This bioluminescence to tissue weight ratio for both the liver and spleen was compared to primary tumor.

- Primary Tumor: $\text{Tumor}\;\text{Burden}/\text{Organ}\left(\mathrm{mg}\right)=\frac{\text{Primary Tumor Bioluminescence}}{\text{Primary Tumor Weight}}.$
- Liver: $\text{Tumor}\;\text{Burden}/\Delta \text{Organ}\left(\mathrm{mg}\right)=\frac{\text{Liver Bioluminescence}}{\left(\text{Cancer Burdened Liver Weight}-\text{Sham Liver Weight}\right)}.$
- Spleen: $\text{Tumor}\;\text{Burden}/\Delta \text{Organ}\left(\mathrm{mg}\right)=\frac{\text{Spleen Bioluminescence}}{\left(\text{Cancer Burdened Spleen Weight}-\text{Sham Spleen Weight}\right)}.$

### 2.9 Metabolic Biomarkers

Blood glucose and ketone (*R* β‐hydroxybutyrate) concentration were measured using Precision Xtra™ Blood Glucose & Ketone Monitoring System (Abbott Laboratories, Abbott Park, IL, USA). Blood lactate concentration was measured using Lactate Plus Lactate Meter (Nova Biomedical). IGF‐1 and insulin were relabeled for blinded analysis and subsequently analyzed using Luminex 100 system (Luminex, Austin, TX, USA) with R&D Systems Mouse 1‐Plex Luminex Assay (R&D Systems, Minneapolis, MN, USA) and Milliplex Mouse Multiplex Kit (Millipore, St. Charles, MO, USA), respectively, using manufacturer's protocol.

### 2.10 Clinical Cachexia Biomarker

At EOL, blood was gathered as previously described and red blood cell count relabeled for blinded analysis via HemoTrue (Heska, Loveland, CO, USA). Remaining whole blood was centrifuged (13,000 rpm, 4 °C, 15 min) down in serum separator tubes, relabeled for blinded analysis (MiniCollect 0.8 ml, Kremsunster, Austria), and analyzed via DRI‐CHEM 7000 (Heska) to determine clinical chemistry concentrations.

### 2.11 Muscle Tissue Collection and Processing

Gastrocnemius muscle tissue was immediately gathered, separated, and flash frozen in liquid nitrogen and stored at −80 °C at harvest. Prior to tissue processing, all tissues were deidentified and relabeled for blinded analysis. For protein and RNA analyses, tissues were removed from −80 °C and crushed using a liquid nitrogen‐cooled mortar and pestle. For protein analysis, ∼30 mg of powdered tissue was placed in 1.7‐ml microcentrifuge tubes containing 500 μl of ice‐cold cell lysis buffer (20 mM Tris‐HCl [pH 7.5], 150 mM NaCl, 1 mM Na_2_EDTA, 1 mM EGTA, 1% Triton [Cell Signaling, Danvers, MA, USA]) pre‐stocked with protease and Tyr/Ser/Thr phosphatase inhibitors (2.5 mM sodium pyrophosphate, 1 mM β‐glycerophosphate, 1 mM Na_3_VO_4_, 1 μg/ml leupeptin). Samples were then homogenized by hand using tight micropestles; insoluble proteins were removed with centrifugation at 500 *g* for 5 min and obtained sample lysates were stored at −80 °C prior to Western blotting and other biochemical assays (described below). For total RNA analysis, ∼15–30 mg of powdered tissue was weighed using an analytical scale with a sensitivity of 0.001 g (Mettler‐Toledo; Columbus, OH, USA). Tissue was then homogenized in 1.7 ml microcentrifuge tubes containing 500 μl of Ribozol (Ameresco; Solon, OH, USA) via micropestle manipulation and RNA isolation was performed per manufacturer recommendations. Total RNA concentrations were then determined in duplicate using a NanoDrop Lite spectrophotometer (Thermo Fisher Scientific), and total RNA per unit muscle weight was used as a surrogate for ribosome density as in past publications.^29^, ^30^

### 2.12 Western Blot Analysis

Whole‐tissue sample lysates obtained through cell lysis buffer processing (described above) were batch process‐assayed for total protein content using a BCA Protein Assay Kit (Thermo Fisher Scientific). Lysates were then prepared for Western blotting using 4 × Laemmli buffer at 1 μg/μl. Following sample preparation, 18 μl samples were loaded onto 4–15% sodium dodecyl sulfate polyacrylamide gels (SDS‐PAGE; Bio‐Rad) and subjected to electrophoresis (180 V for 45–60 min) using premade 1 × SDS‐PAGE running buffer (Ameresco; Framingham, MA, USA) in order of Sham, then Cancer, from animal number 1 to 12. Proteins were then transferred (200 mA for 2 hr) to polyvinylidene difluoride membranes (Bio‐Rad), ponceau‐stained and imaged to ensure equal protein loading between lanes. Membranes were then blocked for 1 hr at room temperature with 5% nonfat milk powder in Tris‐buffered saline with 0.1% Tween‐20 (TBST; Ameresco). Rabbit anti‐mouse pan nuclear factor‐kappa B (NF‐κB)/p65 (1:1,000; cell signaling, catalog #: 8242), rabbit anti‐mouse MuRF‐1 (1:500; Abcam, Cambridge, MA, USA; catalog #: ab172479), rabbit anti‐mouse Atrogin‐1 (1:500; Abcam, catalog #: ab74023), rabbit anti‐mouse Forkhead‐box protein O3a (FOXO3a; 1:500; cell signaling, catalog #: 2497), rabbit anti‐mouse ubiquitin (1:1,000; catalog #: 3933; cell signaling) and rabbit anti‐mouse 20S proteasome core (1:500; Millipore Sigma, Burlingame, MA, USA; catalog #: ST1053) were incubated with membranes overnight at 4 °C in TBST with 5% bovine serum albumin. The following day, membranes were incubated with horseradish peroxidase‐conjugated anti‐rabbit IgG (1:2,000; cell signaling; catalog #: 7074; Danvers, MA, USA) in TBST with 5% bovine serum albumin at room temperature for 1 hr (1:2,000). Membrane development was performed using an enhanced chemiluminescent reagent [Luminata Forte Horseradish Peroxidase (HRP) substrate; EMD Millipore, Billerica, MA, USA], and band densitometry was performed using a gel documentation system and associated densitometry software (UVP, Upland, CA, USA). Densitometry values for all targets were divided by whole‐lane ponceau densities. Density/ponceau were divided by the Sham mean and expressed as relative fold‐change relative to the Sham group.

### 2.13 PCR

Two μg of RNA was reverse transcribed into cDNA for Real‐Time Polymerase Chain Reaction (RT‐PCR) analysis with cDNA synthesis reagents (Quanta Biosciences, Gaithersburg, MD, USA) per the manufacturer's recommendations. RT‐PCR was performed using gene‐specific primers and SYBR green chemistry (Quanta Biosciences). Primer sequences used were as follows: beta‐glucuronidase (housekeeping gene, *HKG*): forward primer 5′‐TCAGCTCTGTGACCGATACG‐3′, reverse primer 5′‐ GCCACAGACCACATCACAAC‐3′; MyoD: forward primer 5′‐ CCTGCCCTCCACATCCTTTT‐3′, reverse primer 5′‐GAAGGGGGAGAGTGGGGTAT‐3′; Atrogin‐1/MAFbx: forward primer 5′‐CCATCCTCTTTCTTGCCCGT‐3′, reverse primer 5′‐ATCACTGTCCAACCTGGCTG‐3′; MuRF‐1: forward primer 5′‐ TGGGACAGATGAGGAGGAGG‐3′, reverse primer 5′‐ TTTACCCTCTGTGGTCACGC‐3′; GPR109a: forward primer 5′‐GATGAAAACATCGCCAAGGT‐3′, reverse primer 5′‐CCTCCAGTCCCAGTTATGGA‐3′; IGF‐1: forward primer 5′‐ACCACCCTGTGACCTCAGTC‐3′, reverse primer 5′‐CTCCTGGAAACCCAGAACAA‐3′. Melt curve analyses confirmed that only one RT‐PCR product was obtained with each primer set. PCR calculations were performed as previously described by our team.^30^ Briefly, 2^ΔCq^ values for each gene of interest at each time point were calculated whereby ΔCq = gene of interest Cq − geometric mean housekeeping gene Cq values. All values for a given mRNA target were then normalized to the Sham mean and expressed as relative fold‐change relative to the Sham group.

### 2.14 20S Proteasome Capacity Assay

Skeletal muscle protein from whole‐tissue sample lysates (40 μg) obtained through cell lysis buffer processing (described above) were batch processed for 20S proteasome activity using commercially available fluorometric kits (Catalog #: APT280; Millipore Sigma) as previously described by our laboratory.^30^ Assay readings are presented as relative fluorometric units normalized to input muscle protein as determined through the BCA assay described above. The average coefficient of variation for all duplicates was 10.7%.

### 2.15 Statistics

GraphPad Prism 7 software was used for all statistical analysis. Parametric tests were performed for all data sets as all groups were considered normally distributed. Unpaired or paired student's *t* tests were performed for the comparison of two groups. One‐way ANOVA with Tukey's multiple comparison post hoc test was performed for more than three comparisons while Fischer Least Significant Difference (LSD) post hoc was used for three comparisons or less. Results were considered significant when *p* < 0.05. Robust regression and outlier removal with coefficient *Q* = 1% was only used prior to cytokine analysis as non‐physiologic/error values were independently indicated.^31^

## 3 Results

### 3.1 VM‐M3 Presents with Progressive Tumor Growth and Spontaneous Systemic Metastases

Clinical reports consistently indicate that cachexia is most prevalent during metastatic disease.^5^, ^7^, ^9^, ^10^, ^11^ To determine tumor growth rate, metastatic progression, and survival specific to cancer and sex in the VM‐M3 model, CA‐M and CA‐F were matched to SH‐M and SH‐F of equivalent sex, bodyweight, and age (*Figures* S1A–S1B). Following implantation of either 1 × 10^6^ VM‐M3 cells expressing a luciferase reporter or PBS vehicle only into VM/Dk mice, tumor growth, and metastatic spread were tracked weekly. CA‐M and CA‐F developed a primary tumor at the implantation site by Week 1, followed by visible metastatic spread to various tissues from tumor origin (*Figure* 1); this was confirmed by *ex vivo* organ and tissue bioluminescence imaging (*Figures* 1C, 1D, 1F, and 1H). Primary tumor weight increased progressively from Week 1 to EOL (*Figure* 1G), with similar metastatic invasion into the liver, spleen, adipose tissue, and ascites fluid (*Figure* 1H) between CA‐M and CA‐F. This is consistent with the progressive nature of systemic metastatic disease, where cachexia and comorbidities are most commonly reported in clinical cancer cachexia.^5^, ^7^, ^9^, ^10^, ^11^ Additionally, survival did not differ between CA‐M and CA‐F (median: 30 and 28 days, respectively, *Figure* 1E; mean: 31.3 ± 1.6 and 32.3 ± 2.4 days, respectively, *Figure* S1C) illustrating similar tumor burden, metastatic spread, and survival between sexes.

### 3.2 VM‐M3 Develops Skeletal Muscle and Adipose Tissue Wasting Not Represented in Bodyweight Measurements

Skeletal muscle and adipose tissue atrophy are common hallmarks of cancer cachexia.^1^, ^2^, ^3^ To determine whether the VM‐M3 model had progressive tissue atrophy, bodyweight was tracked daily during initial analysis and weekly during subsequent analysis as a superficial marker of body composition (*Figure* 2A; *Table* S1). CA‐M and CA‐F gained significantly more bodyweight than Sham groups (*Figure* 2B); however, a characteristic feature of the VM‐M3 model upon presentation of widespread metastatic burden is the accumulation of ascites fluid in the intraperitoneal cavity, which contributes to the elevation in bodyweight. This characteristic is also seen in metastatic patients.^32^ To address this concern, ascites fluid was weighed at EOL to determine its contribution to bodyweight gain. Ascites fluid accounted for 5.5 ± 1.0 g and 6.3 ± 1.4 g in CA‐M and CA‐F bodyweights, respectively (*Figure* 2C), explaining in part, along with tumor burden, the elevations in bodyweight seen in VM‐M3 animals.

Clinically, attributing bodyweight changes to skeletal muscle and/or adipose tissue loss can inaccurately represent the nature of wasting and misguide treatment strategies and “successes.”^33^, ^34^, ^35^ Thus, to directly assess cachexia tissue composition, skeletal muscle, adipose, and tissue weights were gathered at EOL. Upon confirmation of tissue wasting at EOL, follow up week‐by‐week cohort analyses were conducted to determine the temporal/progressive nature of atrophy across tissues and groups. All individual tissues were set to ratio with baseline bodyweight (so not to be influenced by cachexia progression) to allow for appropriate comparison across groups. CA‐M experienced a significant decrease in gastrocnemius and soleus weights starting at Week 2 (−12.0%) and extending to EOL (−19.5%), compared to SH‐M (*Figure* 2D), consistent with the progressive nature of cachectic wasting.^1^ Similar trends were observed in CA‐F, with decreased gastrocnemius and soleus weights at Week 2 (−10.7%; *p* = 0.09), compared to SH‐F (*Figure* 2E), which were significantly decreased within and across groups at EOL (−20.8%). Quadriceps weight was retained in both CA‐M and CA‐F until EOL (*Figure* 2F), indicating temporal and tissue‐specific differences in skeletal muscle wasting which have been observed previously in cachexia models^36^ and other clinical atrophy conditions,^37^ but dissimilar to sex‐specific quadricep atrophy in clinical cachexia.^38^ CA‐M also presented with progressive decreases in intraperitoneal adipose tissue, with significant decreases at Week 3 and EOL within and across groups (*Figure* 2F). Discrepantly, CA‐F retained adipose tissue mass up to Week 3, followed by rapid and complete wasting of adipose tissue between these timepoints. This altered skeletal muscle and adipose tissue composition observed in CA‐F is consistent with previous reports in other female cachexia preclinical models^39^ and patients,^40^ but not others,^38^ and might be explained by inherent hormonal differences influencing skeletal muscle^41^ and adipose tissue.^42^ The data here serve to confirm tissue atrophy and sex‐specific cachectic discrepancies in the VM‐M3 model.

### 3.3 VM‐M3 Develops Prolonged Systemic Inflammation

Inflammation has been reported to drive multiple facets of the cachexia phenotype, including tissue wasting, anorexia, metabolic abnormalities, and tumor progression, among others.^3^, ^43^ To determine if VM‐M3 animals developed systemic inflammation, spleen weight, white blood cell count, and cytokines were measured. Both CA‐M and CA‐F developed splenomegaly (*Figure* 3A); however, increased spleen weight could also be attributed to tumor burden and/or other immunologic initiators.^44^ Thus, to determine whether enlarged spleen weight could be directly attributed to tumor burden alone, a ratio of tumor burden (bioluminescence in photon/s) to cancer‐induced tissue weight changes (Cancer to Sham tissue weight differences) was calculated for the primary tumor, liver, and spleen, as the primary tumor bioluminescence would be directly proportional to increased tumor size and serve as a control ratio. The ratio of tumor burden to cancer‐induced tissue weight differences (*Figure* 3B) was similar between the primary tumor and liver, but dissimilar to spleen, indicating that the change in weight within the liver could be primarily and/or completely attributed to internal organ tumor burden. The spleen weight changes, along with G‐CSF elevations (*Figure* S2B) indicated spleen enlargement was not due to tumor burden but was indicative of a prominent immunologic response.^44^, ^45^ Additionally, white blood cell counts, along with cellular subpopulations (monocytes and granulocytes), were significantly elevated in CA‐M (*Figure* 3C). In CA‐F, however, white blood cell counts were not significantly elevated, although they did show similar trends to CA‐M. Tumor necrosis factor‐α (TNF‐α) and interleukin (IL)‐6, proinflammatory cytokines commonly reported in the cachexia phenotype, were elevated in CA‐M and CA‐F across and within groups (*Figures* 3D and 3E).^3^, ^43^ However, IL‐1β, another proinflammatory cytokine reportedly associated with some cachexia phenotypes, was not significantly altered within or across groups (*Figure* 3F). These inflammatory biomarkers at the organ, cellular, and molecular levels within VM‐M3 animals are indicative of a prolonged systemic inflammatory response and consistent with the cancer cachexia phenotype.^3^, ^43^

### 3.4 VM‐M3 Develops Anorexia, Anemia, Protein Breakdown, Hypoalbuminemia, and Metabolic Derangement

Anorexia, anemia, elevated markers of protein breakdown, hypoalbuminemia, and metabolic derangement remain prominent clinical comorbidities of the full CACS,^2^, ^9^, ^10^, ^11^, ^46^, ^47^, ^48^, ^49^, ^50^ but are often not evaluated in preclinical modeling to determine the potential clinical relevance of these model systems. To determine whether the VM‐M3 model developed the full CACS as clinically presented, all aforementioned clinical comorbidities were evaluated. Food intake was monitored daily to determine the presence of anorexia, a common, contributory, and clinically impactful characteristic of CACS.^10^, ^11^ Both CA‐M and CA‐F developed anorexia, compared to the elevation in food intake seen in both SH‐M and SH‐F (*Figure* 4A). Anemia can be a result of inflammation and/or shifted metabolic demands away from red blood cell production,^44^, ^51^ which can contribute to fatigue and might explain the noticeable lethargy/functional decline in cachexia patients^2^, ^46^ and VM‐M3 animals. CA‐M‐developed anemia as indicated by significantly reduced hemoglobin, hematocrit, and red blood cell count (*Figure* 4B). CA‐F had significant reductions in red blood cell count and trends for decreases in hemoglobin. Blood urea nitrogen and total protein, clinical markers of whole‐body protein kinetics, are commonly elevated and decreased, respectively, in CACS patients.^2^ CA‐M and CA‐F had significantly elevated blood urea nitrogen levels (*Figure* 4B). CA‐M and CA‐F had significantly and non‐significantly reduced total protein levels, respectively. Both biomarkers illustrated increased systemic protein breakdown. Albumin, another critical biomarker commonly reduced in CACS patients, was decreased in VM‐M3 animals (*Figure* 4B). This decrease is hypothesized to be a consequence of shunting of hepatic resources towards acute response proteins during the inflammatory response in CACS^2^, ^46^ and/or nutritional status^52^ and has been linked to higher mortality in cancer, cachexia, and other atrophy diseases.^46^, ^52^, ^53^ CA‐M and CA‐F also developed hypocholesterolemia, without changes in circulating triglycerides or lipase, potentially attributed to inflammation,^54^ disease progression, and splenomegaly^55^ (*Figure* S3).

Blood glucose, *R* β‐hydroxybutyrate, and lactate were also measured weekly to further evaluate metabolic alterations associated with the model. Blood glucose did not change, except for a significant decrease from baseline to Week 1 in CA‐M (*Figure* 4C); however, this did not exclude the possibility that metabolic changes were occurring while the serum metabolites remained within the homeostatic range, as elevated glucose turnover has been reported in cachexia patients.^47^, ^48^, ^50^ Blood *R* β‐hydroxybutyrate did not differ significantly between Sham and Cancer groups for male and female mice, except for Week 3 for males and Week 1 for females. While limited female cohort availability did not allow for assessment across both sexes, follow‐up analysis indicated that blood lactate was significantly elevated in CA‐M (*Figure* 4C), indicating that metabolic alterations were present likely via the aerobic fermentation pathway (systemically and/or tumor driven) that may have contributed to increased metabolic inefficiency.^47^, ^49^, ^50^ Collectively, these observations in circulating biomarkers were not explained by altered hydration status (*Figure* S4). Taken together, VM‐M3 animals demonstrated comorbid anorexia, anemia, elevated markers of protein breakdown, hypoalbuminemia, and metabolic derangement, modeling the comprehensive CACS as clinically described.

### 3.5 IGF‐1/Insulin‐FOXO3a‐Ubiquitin Proteasome Pathway is Activated in VM‐M3 Skeletal Muscle

Multiple cachexia atrophy mechanisms have been identified in rodent modeling and emergent evidence indicates that the ubiquitin proteasome degradation pathway is a prominent contributor to skeletal muscle atrophy.^3^, ^56^, ^57^ To determine the mechanism of skeletal muscle atrophy in the VM‐M3 model of CACS, follow‐up analyses were conducted in CA‐M, and activation status of the ubiquitin proteasome pathway was examined. Results confirmed muscle protein poly‐ubiquitination activation (*Figure* 5A; *Figure* S5D), a critical step in the irreversible process of protein degradation.^58^ Specifically, 26S proteasome quantity and capacity were quantified via 20S proteasome core western blotting and fluorescence LLYT peptide quantitation of muscle tissue lysates, respectively. Neither quantity (*Figures* 5B; *Figure* S5E) nor capacity (*Figure* 5B) of the 26S proteasome were altered, indicating atrophy likely occurred via upregulation of muscle protein poly‐ubiquitination.

TNF‐α has also been shown to play a direct role in skeletal muscle atrophy by upregulating NF‐κB and subsequently ubiquitin proteasome activity.^59^, ^60^ Following prior confirmation of TNF‐α elevation (*Figure* 3D), NF‐κB activation was explored in VM‐M3 skeletal muscle to determine which upstream pathway was associated with ubiquitination. Nuclear fraction versus total NF‐κB (65 kDa) protein analysis revealed no significant differences between CA‐M and SH‐M (*Figure* 5C; *Figures* S5F and S5G); however, NF‐κB is temporally regulated, and analysis may have missed the activation timepoint.^61^ Thus, mRNA expression of MyoD, MuRF‐1, Atrogin‐1, and GRP109a as well as protein levels of MuRF‐1 and Atrogin‐1 were analyzed as surrogates of NF‐κB activation. MyoD expression drives myogenesis in skeletal muscle, but activation of NF‐κB is known to directly induce the degradation of MyoD mRNA.^62^ Results indicated MyoD mRNA was unchanged in CA‐M (*Figure* 5D). Additionally, MuRF‐1 and Atrogin‐1 are skeletal muscle‐specific E3 ligases which can be upregulated via NF‐κB activation; however, both mRNA and protein levels were similar between conditions (*Figures* 5E and 5F; *Figure* S5H and S5I). mRNA levels for GPR109a, a receptor protein known to be upregulated by NF‐κB activation and subsequently induce negative feedback upon NF‐κB pathway activation,^63^, ^64^ revealed no differences between conditions either (*Figure* S5A). Interestingly, total RNA levels (a surrogate of ribosome density) and nuclear fraction protein levels were significantly reduced in the VM‐M3 skeletal muscle (*Figures* S5B and S5C) further suggesting these animals were in a catabolic state. TNF‐α is known to chronically activate NF‐κB. Given that all of the aforementioned markers indicated no differences between conditions regarding NF‐κB pathway activity, serum levels of a reported counter‐regulator of TNF‐α‐induced NF‐κB activation in muscle, IL‐10, was explored.^65^ Serum IL‐10 was elevated, potentially explaining our NF‐κB results (*Figure* 5G) despite the increases in TNF‐α (*Figure* 3D) and poly‐ubiquitinated protein levels (*Figure* 5A).

Another upstream activator of the ubiquitin proteasome pathway is via IGF‐1/insulin, FOXO3a, and Atrogin‐1 signaling.^56^, ^66^ Serum IGF‐1 and insulin levels were analyzed in VM‐M3 animals to determine if this upstream mechanism was associated with muscle protein poly‐ubiquitination. Quantification of serum IGF‐1 and insulin revealed 26‐fold and 4.6‐fold downregulation of both anabolic hormones, respectively (*Figure* 5H). Quantification of nuclear fraction FOXO3a revealed a 2.3‐fold increase in VM‐M3 animals (*Figure* 5I). Collectively, these results suggest that VM‐M3 skeletal muscle atrophy may occur through a reduction in serum IGF‐1/insulin and a subsequent increase in FOXO3a activation resulting in an increase in muscle protein poly‐ubiquitination; all of which was independent of TNF‐α‐induced NF‐κB activation.

### 3.6 Ketone Diester Mitigates Comorbidities, Tumor Burden, and Skeletal Muscle Atrophy in Cancer Anorexia Cachexia Syndrome

Existing evidence implicates IGF‐1/insulin, FOXO3a, and the ubiquitin proteasome pathway in muscle homeostasis and numerous atrophy conditions,^56^, ^57^ including nutrient deprivation.^66^, ^67^ Interestingly, patients undergoing extreme nutrient deprivation (i.e., prolonged fasting/anorexia) upregulate endogenous ketone body production, which is hypothesized to allow for prolonged survival via a protective and progressive metabolic adaptation that attenuates muscle atrophy.^15^, ^16^ Previously, dietary restrictions and/or infusion‐induced elevations of ketone bodies limited the exploration and clinical translation of therapeutic ketosis; however, a novel, orally ingestible exogenous KDE may be a viable therapeutic strategy. This KDE was assessed to determine its effect on anorexia, systemic metabolism, tumor and metastatic burden, and skeletal muscle catabolism in multifactorial CACS modeling.

To determine whether KDE could alter systemic metabolism, blood metabolites were measured at baseline following a standard diet and after a 4 day transition to a standard diet supplemented with 20% KDE, 25% KDE, or 30% KDE. Results indicated 20% KDE and 30% KDE caused significant elevations in blood ketone levels, with 30% KDE inducing the highest ketone elevations (*Figure* S6C). All KDE groups exhibited significant reductions in blood glucose levels, with 30% KDE inducing the greatest reduction in glucose. A second study was initiated to expand upon these findings, as prior work revealed that exogenous administration of ketone bodies may reduce ad libitum food intake^68^, ^69^ and confound evaluation of KDE effect on catabolism. To control for this effect, food palatability was increased by incrementally integrating the KDE into the diet (5%/day) over a 7‐day period to achieve 30% KDE. The 30% KDE was well‐tolerated as indicated by bodyweight maintenance (*Figure* S6B), and since 30% KDE caused the greatest alteration in systemic metabolism, mirroring the metabolic alterations seen during extreme nutrient deprivation,^15^ 30% KDE was administered in VM‐M3 CACS (KDE + VM‐M3) utilizing intraperitoneal implantation to determine whether KDE would alter the course of CACS‐induced atrophy compared to VM‐M3 CACS alone (VM‐M3) and PBS only (Sham). Food intake was tracked daily to determine if KDE administration altered the anorexic phenotype. VM‐M3 experienced a reduction in food intake (*Figure* 6A); however, animals in the KDE + VM‐M3 demonstrated attenuation of the predicted anorexic phenotype in this CACS model (*Figure* 6A). To determine whether KDE could alter metabolism in CACS, blood ketones, glucose, and lactate were measured weekly. KDE induced sustained elevations in circulating ketones and reductions in blood glucose in KDE + VM‐M3 compared to both VM‐M3 and Sham (*Figure* 6B), illustrating KDE‐induced alterations in CACS systemic metabolism. No difference was found between KDE + VM‐M3 and VM‐M3 for blood lactate (*Figure* 6B), potentially indicating similar tumor or inflammation‐induced aerobic fermentation.^70^, ^71^ Thus, to determine if KDE altered tumor burden, *in* *vivo* bioluminescence imaging was analyzed weekly. Interestingly, KDE + VM‐M3 demonstrated non‐significant reductions in whole animal tumor burden (*Figure* 6C). Additionally, ascites fluid, a surrogate marker of metastatic spread,^32^ was significantly reduced in KDE + VM‐M3 compared to VM‐M3 (*Figure* 6D), indicating KDE‐induced reductions in markers of tumor burden.

To determine if KDE altered body composition and whether this was through alteration in model‐specific catabolic drivers, bodyweight was tracked daily, while serum IGF‐1 and insulin as well as relative masses for cardiac tissue, adipose tissue, and skeletal muscle tissue were evaluated 3 weeks post‐implantation, prior to EOL. KDE + VM‐M3 had a lower bodyweight compared to VM‐M3 and Sham (*Figure* 6E); however, as both ascites fluid and tumor burden confound bodyweight in VM‐M3 animals, bodyweight alone could not reliably indicate tissue atrophy in the VM‐M3 model of CACS. KDE + VM‐M3 and VM‐M3 both demonstrated significantly reduced serum IGF‐1 and insulin, demonstrating that KDE did not alter circulating anabolic hormone levels (*Figure* 6F). Cardiac tissue was found to be unaltered by CACS or across groups (*Figure* 6G). Additionally, adipose tissue atrophied to equivalent levels in KDE + VM‐M3 and VM‐M3, indicating KDE was unable to alter adipose tissue catabolism. However, the KDE did attenuate muscle atrophy across numerous skeletal muscle tissues (*Figure* 6I). Taken together, the KDE was well tolerated, attenuated anorexia, altered systemic metabolism, attenuated indices of tumor burden, and reduced skeletal muscle atrophy without changing circulating IGF‐1 and insulin levels, illustrating a unique and multifaceted anti‐CACS therapy.

### 3.7 Ketone Diester Mitigates Bodyweight Loss and Comorbidities in Inflammation‐Induced Atrophy

**Figure 7:**
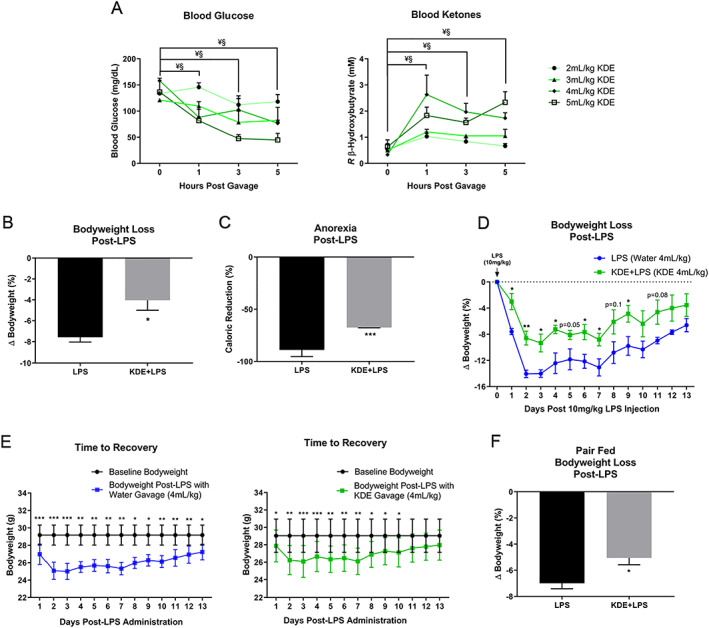
**Ketone Diester Mitigates Bodyweight Loss and Comorbidities in Inflammation‐Induced Atrophy**. (**A**) Blood ketones and glucose were quantified for ketone diester (KDE) at 2 ml/kg (*n* = 3), 3 ml/kg (*n* = 2), 4 ml/kg (*n* = 3), 5 ml/kg (*n* = 3) via enzyme interaction. KDE at 4 ml/kg demonstrated shifts in system metabolism. Data: Experiment 5a. (**B**) Percentage bodyweight loss 1‐day post administration of 10 mg/kg lipopolysaccharide with either 4‐ml water (LPS, *n* = 4) or 4 ml/kg KDE (KDE + LPS, *n* = 5) gavage. Data: Experiment 5b. (**C**) Percentage caloric restriction 1‐day post LPS (*n* = 4) or KDE + LPS (*n* = 5). Data: Experiment 5b. (**D**) Percentage bodyweight loss tracked for 13 days following LPS (*n* = 4) or KDE + LPS (*n* = 5). Data: Experiment 5b. (**E**) Time to recovery as time taken to return to baseline bodyweight following LPS (*n* = 4) or KDE + LPS (*n* = 5). Data: Experiment 5b. (**F**) Percentage bodyweight loss in pair‐fed animals 1 day post LPS (*n* = 4) or KDE + LPS (*n* = 4). Data: Experiment 5c. **Data Information**: Within group differences compared to time point “0” were analyzed with one‐way ANOVA with Fischer LSD post hoc for ≤3 comparisons (A). Difference across groups were analyzed unpaired (B‐D,F). Differences across time point were analyzed with paired *t* test (E). Colors (D‐E): KDE + LPS, green; LPS, blue. Data are mean ± *SEM*. 4 ml/kg different from time point “0” ^**§**^ ***P*** < 0.05, 5 ml/kg different from time point “0” ^**¥**^ ***P*** < 0.05 (A). ^**ns**^ ***P*** > 0.05, * ***P*** < 0.05, ** ***P*** < 0.01, *** ***P*** < 0.001 (B‐F).

Reductions in anorexia, tumor burden, and skeletal muscle atrophy all present ideal outcomes in the clinical environment of CACS. It should be noted, however, that altered anorexia and tumor burden can confound interpretation of the direct effects of the KDE on skeletal muscle atrophy. To determine direct effects of KDE on catabolism when controlling for confounding variables of cancer and anorexia, KDE was evaluated in an inflammation‐induced atrophy environment of LPS‐induced sepsis, which has been shown to produce an overlapping multifactorial atrophy environment of low IGF‐1/insulin, systemic inflammation, anorexia, anemia, hypoalbuminemia, metabolic derangement, and upregulated ubiquitin proteasome signaling.^2^ Due to the rapid nature of LPS‐induced atrophy, various dosages of KDE were gavaged to determine their ability to rapidly shift systemic metabolism. Both 4 ml/kg and 5 ml/kg KDE dosages resulted in significant and rapid reductions in blood glucose and elevations in blood ketones (*Figure* 7A). To determine KDE's effect on cancer‐independent catabolism, a maximal nonfatal LPS dose (10 mg/kg) was administered, followed by a single 4 ml/kg water (LPS) or KDE (KDE + LPS) gavage. KDE attenuated bodyweight loss 47% within the first 24‐hr post‐LPS administration (*Figure* 7B). Consistent with what was found in CACS, KDE also significantly reduced anorexic symptoms (*Figure* 7C).

Animals were followed over a 13‐day period to determine chronic effects of KDE. KDE significantly attenuated chronic LPS‐induced bodyweight loss (*Figure* 7D). To determine whether KDE would alter length of recovery time from a maximal nonfatal LPS dose, bodyweight was assessed over the 13‐day time period to determine length of time before animals returned to baseline bodyweight. KDE + LPS recovered within 10 days, while LPS did not recover within the 13‐day time period (*Figure* 7E). While this illustrates that a single KDE administration could rapidly alter systemic metabolism and attenuate catabolism and comorbid symptoms, reducing anorexia can potentially confound catabolism. To determine if KDE attenuated catabolism in post‐LPS administration when controlling for anorexia, animals were pair‐fed and administered a single 4 ml/kg gavage. Pair‐fed KDE + LPS significantly reduced bodyweight loss 28% compared to LPS group (*Figure* 7F), indicating KDE attenuated catabolism independent of food intake. Taken together, the KDE attenuated anorexia and catabolism in a cancer‐independent inflammatory‐atrophy environment when comorbidities were controlled, suggesting a direct anti‐catabolic effect of KDE in these multifactorial catabolic environments.

## 4 Discussion

An inability to model the clinically reported environment of progressive metastatic CACS and discover effective treatments that mitigate both muscle atrophy and comorbid symptoms has impeded clinical advancements in the most commonly affected patient population, advanced metastatic cancer patients.^5^, ^7^, ^9^, ^10^, ^11^ Our results demonstrate that the VM‐M3 model replicates the progressive and spontaneous nature of metastatic disease which facilitates the development of the full clinical CACS environment via progressive wasting of skeletal muscle with observed alterations in IGF‐1/insulin, FOXO3a, and ubiquitin proteasome degradation pathway, along with adipose tissue wasting, systemic inflammation, anorexia, anemia, hypoalbuminemia, elevated protein breakdown, and metabolic derangement with sex‐specific discrepancies. Additionally, our work demonstrates that the administration of a non‐toxic KDE added to a standard diet was well tolerated, shifted systemic metabolism, mitigated comorbid symptoms, attenuated tumor burden indices, and reduced catabolism in both the progressive metastatic CACS and cancer‐independent septic/inflammatory atrophy environments. These results illustrate that the KDE is a unique, novel, and multifaceted anti‐CACS therapeutic with direct anti‐catabolic effects in atrophy environments.

The VM‐M3 model presents a unique metastatic model in which subcutaneously implanted VM‐M3 luciferase‐expressing cells can develop a primary tumor, spontaneously leave the primary tumor site, enter the circulation, and produce distant metastases that can be monitored by bioluminescence imaging. Intraperitoneal implantation of VM‐M3 cells also induced similar systemic metastases, progressive tumor burden, and CACS phenotype to subcutaneous implantation illustrating a consistent modeling system (*Table* S2). This is distinct from other models which require intravenous implantation or the assistance of surgical resection of the primary tumor to induce metastases.^7^ The reliable, reproducible, and logistically feasible nature of this metastatic model^13^, ^14^ may present numerous experimental advantages over emergent genetically engineered modeling systems which often require lengthy and costly experimental designs due to heterogeneous tumor onset and growth, metastatic progression, and cachexia occurrence. Additionally, metastases can directly disrupt cancer‐burdened tissue and indirectly disrupt noncancerous tissue through secretory factors or alterations in host‐tissue response,^12^ further highlighting the need to model the complex metastatic CACS.

Among the hallmark characteristics of cachexia is the atrophy of skeletal muscle and often adipose tissue,^1^, ^2^, ^3^ with bodyweight being most commonly used clinically as a surrogate marker for both due to ease of assessment. It is important to note, however, that tissues can atrophy and/or hypertrophy in an asymmetric manner, highlighted in cancer patients with sarcopenia obesity and in cachexia trials using appetite stimulants, where it has been reported that adipose tissue can increase while skeletal muscle progressively atrophies.^33^, ^34^, ^35^ Similarly, we demonstrated the nonspecificity of bodyweight as a determinant for cachexia status, while tissue specific assessment of wasting did reveal progressive skeletal muscle wasting, the hallmark characteristic of cachexia.^1^, ^2^, ^3^ Interestingly, while hind limb weights atrophied in a progressive manner, we did not observe decreases in quadriceps tissue until after Week 3. This preferential retention in quadricep muscle mass over gastrocnemius and soleus has also been reported in the C26 cachexia rodent model^36^ and in patients with disuse atrophy.^37^ This may be explained by gene expression differences across muscle groups^72^ as epigenetic modulation of gene expression has been shown to regulate wasting in cachexia, disuse atrophy, and nutrient deprivation.^67^, ^73^ While we observed quadricep atrophy across sexes, sex‐specific quadricep wasting has been previously reported,^38^ highlighting a reported need for further sex‐specific analysis in clinical cohorts.^74^ While skeletal muscle wasting is central to the disease, adipose tissue wasting has gained considerable attention, as recent reports have demonstrated that adipose tissue wasting may regulate skeletal muscle wasting in several cachexia models.^75^ We demonstrate that VM‐M3 males had progressive adipose tissue wasting, while VM‐M3 females demonstrated sex‐specific retention of adipose tissue until after Week 3. Previous reports confirm differential degree and timing of tissue wasting^39^, ^40^ and function^76^ between males and females, hypothesized^76^ or explained by hormonal differences.^39^

While metastasis and tissue wasting are important components of CACS, accompanying systemic inflammation, anorexia, anemia, elevated protein breakdown, hypoalbuminemia, and metabolic derangement demonstrate the full multifactorial CACS.^1^, ^2^, ^3^, ^9^, ^10^, ^11^, ^43^, ^46^, ^47^, ^48^, ^49^, ^50^ Inflammation has been commonly reported to play roles in multiple aspects of the CACS wasting scenario including tissue wasting, anorexia, metabolic abnormalities, tumor progression, among others.^3^, ^43^ We observed progressive systemic inflammation across metrics of spleen enlargement, white blood cell elevation, and augmented pro‐inflammatory cytokines. Anorexia is a common, contributory, and clinically impactful side effect of CACS.^2^, ^10^, ^11^ Both cachexia and anorexia can be mechanistically driven by a pro‐inflammatory state,^43^ which were simultaneously observed in the VM‐M3 model. Finally, we demonstrated that the VM‐M3 model had the comprehensive clinical biomarkers observed in cachexia patients that are often unevaluated in rodent model systems, including anemia, metrics of protein breakdown, hypoalbuminemia, and metabolic derangement. This is salient as many mechanisms and therapeutic strategies proposed for CACS are largely dependent on modeling systems known to not recapitulate or remain unevaluated for the full CACS.

Multiple driving mechanisms of skeletal muscle atrophy have been proposed and explored for cachexia,^3^ yet limited patient data lead to many unanswered questions related to the underlying drivers at the skeletal muscle level.^6^ However, emerging evidence from rodent models indicates that the ubiquitin proteasome degradation pathway is a prominent contributor to skeletal muscle atrophy.^3^, ^56^, ^57^ We observed elevated serum TNF‐α and skeletal muscle protein poly‐ubiquitination, without alterations in acute or chronic markers suggestive of NF‐κB activation which could be explained by an observed elevation in IL‐10 serum levels.^65^ We did, however, note significantly reduced serum IGF‐1 and insulin levels along with elevated nuclear FOXO3a levels in skeletal muscle; the latter being an established upstream modulator of the ubiquitin proteasome pathway.^56^, ^66^ Importantly, IGF‐1/insulin signaling has an established role in muscle homeostasis and alterations in pathway modulators and/or signaling components have been observed in patients across multiple atrophy environments including cachexia, nutrient deprivation/anorexia, sepsis, diabetes, sarcopenia, amongst others, demonstrating a clinically relevant mechanism in atrophy‐based disease.^3^, ^56^, ^57^

While the VM‐M3 model serves as a robust and comprehensive modeling system for CACS, there is currently no effective treatment for this multifaceted disease. KDE is a non‐toxic synthetic exogenous ketone compound composed of a *R/S* 1,3‐butanediol backbone esterified to two acetoacetates. Upon oral administration, KDE increases circulating levels of the ketone bodies β‐hydroxybutyrate and acetoacetate, while decreasing blood glucose in a dose‐dependent manner without the barriers of whole lifestyle/dietary changes or IV infusion.^77^, ^78^ We observed elevations in circulating ketone bodies and decreases in blood glucose in a dose‐dependent manner when administered chronically via food and acutely via oral gavage in the context of a standard diet. This presents a major step toward the clinical advancement of ketone therapeutics as fasting‐induced ketone elevations and glucose reductions in cachexia patients are contraindicated, due to a fear that nutrient deprivation will exacerbate tissue atrophy. While the very low carbohydrate ketogenic diet has overlapping metabolic effects and circumvents nutrient deprivation, major dietary modifications are difficult for some patients to sustain,^79^ and compliance can be further complicated by disease. Moreover, while direct IV infusion of metabolites into circulation allows for dose‐dependent elevations in circulating ketone bodies without altering diet, this is limited to the clinical or research environment. Thus, KDE provides an orally consumable and well‐tolerated method for modulating systemic metabolism without dietary restriction or direct circulatory infusion.

Tumor burden and metastatic spread directly impact cachexia risk and progression. Prior work has demonstrated that a very low carbohydrate ketogenic diet can attenuate cancer burden across various preclinical cancer models.^23^, ^80^ Ketone bodies have also been demonstrated to induce direct anticancer effects which have been proposed to occur via alterations in energetic metabolism, oxidative stress, inflammation, and/or epigenetic regulation.^14^, ^23^, ^80^, ^81^ Here we demonstrate that chronic KDE administration in the context of a standard diet mitigated indices of tumor burden. Interestingly, while we would expect caloric restriction to induce an antitumor response, KDE antitumor response was present even though comorbid anorexia (involuntary caloric restriction) was mitigated in the KDE group. Seemingly contrary to the KDE anti‐anorexic effects presented here are the ketone‐induced reduction in food intake previously reported.^68^, ^69^ This was achieved through the alteration of standard diet palatability and a stepwise integration of KDE to mitigate this confounder in non‐diseased animals. Additionally, we demonstrated that the KDE mitigated anorexia in both the CACS and LPS/septic anorexic environments. Given the established role of inflammation in anorexia^43^ and the reported anti‐inflammatory effects of KDE,^25^, ^26^ it is possible that ketones may mitigate anorexia via alterations in systemic inflammation in both CACS and LPS/sepsis.

Atrophy/catabolism is the hallmark characteristic of CACS, and ketone bodies have been shown to be associated with or directly reduce metrics of protein breakdown across various populations following nutrient deprivation,^15^, ^16^ acute IV infusion^16^, ^17^, ^18^ or through in vitro analysis.^20^, ^21^ However, the chronic effects of ketone bodies on the principle outcome, atrophy/catabolism, have not been determined, nor has the efficacy of orally administered exogenous ketone bodies been tested across various multifaceted atrophy environments. Here we demonstrate that administration of oral exogenous ketone bodies mitigated catabolism in two multifactorial and overlapping inflammatory environments. First, KDE attenuated muscle atrophy in CACS without altered input from upstream anabolic hormones deemed mechanistically involved in tissue wasting. Second, KDE mitigated LPS/sepsis catabolism even when controlling for comorbid food intake/anorexia. Mechanistically, patients exhibited reduced IGF‐1/insulin, stably decreased glucose, and increased ketone bodies during prolonged fasting‐induced atrophy.^15^, ^16^ These studies also uncovered that as ketone bodies become elevated, metrics of protein breakdown progressively decreased along with a threefold to sixfold reduction in amino acid efflux from skeletal muscle, suggesting a direct ketone‐induced effect on protein turnover. Additionally, recent work in patients with LPS/sepsis demonstrated that direct IV infusion of ketone bodies induced a potent acute anti‐catabolic response in the skeletal muscle, even when controlling for the potential confounding effects of GPR109a (HCAR2) signaling on NF‐κB.^17^, ^27^ Importantly, fasting,^15^, ^66^ LPS/sepsis,^82^ and CACS^3^, ^83^ have all been shown to reduce IGF‐1/insulin signaling. This overlapping atrophy signaling in our work and others indicating that ketone bodies attenuate catabolism in all three conditions,^15^, ^16^, ^17^ even when controlling for confounding GPR109a/NF‐κB signaling and circulating IGF‐1/insulin levels, suggests other possible mechanisms. Ketone‐induced histone acetylation via Classes I and II histone deacetylase (HDAC) inhibition is one possible anti‐catabolic mechanism as (a) Classes I and II HDAC‐induced atrophy occurs across multiple catabolic states^67^, ^84^, ^85^; (b) inhibition of Classes I and II HDACs and the subsequent histone acetylation in skeletal muscle is sufficient to inhibit skeletal muscle atrophy via the restriction of FOXO3a nuclear accumulation^67^; (c) genetic deletion of HDAC4 attenuates activations of ubiquitin proteasome degradation in multiple models of atrophy^86^, ^87^; (d) ketone bodies inhibit Classes I and II HDACs and promote histone acetylation in many tissues,^88^ including skeletal muscle^22^; (e) ketone bodies elicit anti‐atrophy effects across multiple catabolic environments.^15^, ^16^, ^17^, ^20^, ^21^, ^27^ However, there is also evidence that FOXO3a levels increase with diet‐induced ketone elevations.^22^, ^88^ Still, neither analysis indicated FOXO3a skeletal muscle nuclear localization. Additionally, FOXO3a has consistently been shown to induce atrophy in skeletal muscle, which contradicts work demonstrating the anti‐atrophy effect of ketone bodies in environments where IGF‐1/insulin signaling is commonly suppressed.^15^, ^16^, ^17^ However, ketone bodies have been demonstrated to have other anti‐catabolic mechanisms which cannot be ruled out in the present analysis.^27^ Thus, KDE‐induced inhibition of Classes I and II HDAC, the subsequent histone acetylation, and efflux of nuclear FOXO3a, along with regulatory capacity across catabolic, synthetic, and metabolic pathways, present potential anti‐atrophy mechanism(s) which should be examined in future analysis. Additionally, while STAT3‐ and autophagy‐induced atrophy signaling remain unexplored in the current analysis, the observed elevations in serum IL‐6 and skeletal muscle FOXO3a nuclear localization indicates that we cannot exclude these potentially contributory pathways.

While our work and others suggest ketone bodies have a pluripotent therapeutic role in wasting environments, the ability of the KDE to induce these effects without altering circulating anabolic hormones may have important clinical relevance. First, various attempts to inhibit FOXO3a and ubiquitin proteasome degradation in the inflammatory environment via elevations in anabolic hormones have not proven efficacious.^82^, ^89^ This is likely due to skeletal muscle resistance to IGF‐1/insulin signaling in these inflammatory environments.^47^, ^90^, ^91^, ^92^ While inflammation‐induced IGF‐1/insulin resistance is not fully understood, multiple reports indicate this may be occurring at the receptor level.^90^, ^91^ Interestingly, our work demonstrated an anti‐catabolic effect of ketone bodies across low IGF‐1/insulin signaling and pro‐inflammatory environments demonstrating that ketones may present a modulatory tool for this catabolic pathway by circumventing previous therapeutic resistance. Secondly, discussion has emerged around the proposed dichotomy of IGF‐1 on skeletal muscle mass, cancer growth, and metastasis,^93^ and longevity.^57^ Thus, ketone bodies' ability to promote optimal muscle mass while mitigating perceived risk for adverse health or disease outcomes is a finding worth future clinical exploration. Beyond tissue atrophy/catabolism, ketone bodies have been demonstrated to directly regulate metabolism, reduce oxidative stress, attenuate inflammation, and regulate epigenetics, amongst other effects.^23^, ^24^, ^25^, ^26^ Consequently, ketone bodies may help support improved patient outcomes across various multifaceted diseases including cancer, CACS, sepsis, amongst others, where atrophy/catabolism is only one component compromising patient outcomes.

Taken together, this work demonstrates a comprehensive, metastatic, and progressive model of CACS with sex‐specific variation, and a non‐toxic exogenous ketone therapy with metabolic, anti‐catabolic, anti‐anorexic, and antitumor therapeutic effects across various multifaceted atrophy/catabolic environments. Future studies are warranted to further investigate other mechanisms through which the KDE attenuates atrophy and comorbidities, determine optimal KDE administration protocol, evaluate potential synergistic therapeutic strategies to optimize therapeutic effect, and most notably, determine whether KDE can be a supportive nutritional therapeutic in clinical CACS and inflammatory atrophy environments.

## Author Contribution

A.P.K. conceptualized, developed, and conducted experiments and analysis, and developed original manuscript draft. A.M.P., N.P.W., D.P.D., and M.D.R. provided experimental input. J.M.D., N.P.W., and M.A.S. assisted with *in vivo* data collected. M.D.R., M.A.S., M.A.R., P.A.R., and C.D.F. assisted with *ex vivo* muscle analysis. D.P.D. and A.M.P. acquired funding and resources. All authors reviewed and approved final manuscript draft.

## Disclosure

At the time of this publication: D.P.D. and A.M.P. are inventors on the following patents: Dominic P. D'Agostino; Angela M. Poff; Patrick Arnold; “Targeting Cancer with Metabolic Therapy and Hyperbaric Oxygen” (Patent Number: 9801903). D.P.D. is the owner of Ketone Technologies LLC, which does consulting and public speaking events. A.M.P. is a scientific advisor to Pruvit Ventures and owner of Poff Medical Consulting and Communications, LLC and Metabolic Health Initiative, LLC. D.P.D., A.M.P., and A.P.K. are inventors on pending patent “Compositions and Methods for Weight Loss Maintenance.” A.P.K. and D.P.D. are inventor on pending patent “Prevention of Muscle Wasting with Ketone Supplementation.” At the time of this publication, pending patents were still under review. Should patents become accepted and royalties ever accrue, A.M.P., A.P.K., and D.P.D. will receive a share under the patent terms prescribed by the University of South Florida. All other authors do not have financial or other relationships that may be perceived as leading to conflict of interest. All authors have approved the final version of this article.

## Supporting information


**Data S1**. Supporting information

**Table S1**. Experimental Design Experimental Design reference table for main experiments. Data information: Abbreviations: *R* βHB, *R* β‐Hydroxybutyrate; SH‐M, Sham Males; CA‐M, Cancer Males; SH‐F, Sham Females; CA‐F, Cancer Females; EOL, End of Life; VM‐M3, VM‐M3 Mouse Model of Systemic Metastasis; KDE, Ketone Diester; LPS, Lipopolysaccharide/Endotoxin. *= One unexplained animal death immediately postinoculation.
**Table S2**. VM‐M3 Subcutaneous Versus Intraperitoneal Implantation. Overlapping phenotype observed with 1×10^6^ VM‐M3 subcutaneous and intraperitoneal implantation. Data information: Abbreviations: CA‐M, Cancer Males; SH‐M, Sham Males. Red line indicates region of interest (ROI). Color Scaling: Radiance (photons/sec/cm^2^/sr).
**Table S3**. Ketone Diester Physical Properties Physical properties reference table for *R/S* 1,3‐Butanediol Acetoacetate Diester. Data information: Abbreviations: GC/MS, Gas Chromatography Mass Spectrum Analysis.
**Table S4**. Ketone Diester Preparation and Analysis. Preparation and confirmation analysis for *R/S* 1,3‐Butanediol Acetoacetate Diester chemical synthesis. Data information: Abbreviations: GRAS, Food and Drug Administration Generally Recognized as Safe; GC‐FID, Gas Chromatography Flame‐Ionization Detection.
**Figure S1**. Baseline Sex, Bodyweight, and Age Controlled with Similar Survival in Males and Females. A Sham Males (SH‐M), Cancer Males (CA‐M), Sham Females (SH‐F), Cancer Females (CA‐F) bodyweight (SH‐M, *n*=20; CA‐M, *n*=20; SH‐F, *n*=18; CA‐F, *n*=18), age (SH‐M, *n*=20; CA‐M, *n*=20; SH‐F, *n*=18; CA‐F, *n*=18), and daily food intake (SH‐M, *n*=12; CA‐M, *n*=12; SH‐F, *n*=11; CA‐F, *n*=12) were matched at baseline. Data: Bodyweight and Age, Experiment 1a&b; Food Intake, Experiment 1a. B Sham Males (SH‐M) week 1 (*n*=4), SH‐M week 2 (*n*=4), SH‐M week 3 (*n*=5), Cancer Males (CA‐M) week 1 (*n*=4), CA‐M week 2 (*n*=4), CA‐M week 3 (*n*=5), SH‐F week 1 (*n*=5), SH‐F week 2 (*n*=5), and SH‐F week 3 (*n*=6), Cancer Females (CA‐F) week 1 (*n*=5), CA‐F week 2 (*n*=5), CA‐F week 3 (*n*=6) were similar in bodyweight and age, although females tended to smaller. No significant differences in food intake were observed between CA‐F or SH‐F except between SH‐F week 3 and CA‐F week 3. Differences were observed across male groups and CA‐F week 2 & 3. Data: Experiment 2. C Mean Survival for CA‐M (*n*=20, 31.3 days) and CA‐F (*n*=18, 32.3 days). Data: EOL, Experiment 1a&b. Data information: Differences across groups at each timepoint were analyzed with One‐Way ANOVA with Tukey's post‐hoc (A,B). Differences across groups were analyzed with unpaired t‐test (C). Data are mean ± SEM. ^ns^ *P*>0.05, *P<0.05, **P<0.01, ****P<0.0001.
**Figure S2**. VM‐M3 Develop Prolonged Systemic Inflammation. A Liver weights as a ratio baseline bodyweight for Sham Males (SH‐M), Cancer Males (CA‐M) (M week 1, *n*=4; week 2, *n*=4; week 3, *n*=5; end of life, EOL, *n*=20), Sham Females (SH‐F), and Cancer Females (CA‐F) (F week 1, *n*=5; week 2, *n*=5; week 3, *n*=6; EOL, *n*=17). Data: Week 1‐3, Experiment 2; EOL, Experiment 1a&b. B Cytokines quantification via Luminex fluorophore intensity analysis (*n*=8‐12/group/timepoint). Data: Week 1‐3, Experiment 2; EOL, Experiment 1a. Data information: Within group differences across time were analyzed with One‐Way ANOVA with Tukey's posthoc with >3 comparisons (A) and Fischer LSD post‐hoc for ≤3 comparisons (B). Differences across groups at each timepoint were analyzed with unpaired t‐test. (A,B) Prior to ANOVA cytokine analysis, robust regression and outlier removal (ROUT) with coefficient Q=1% was used as non‐physiologic/error values were detected (B). Abbreviations: IP‐10, IFN‐γ‐Inducible Protein 10; KC, Keratinocyte‐Derived Cytokine; MIG, Monokine Induced by IFN‐γ. Colors (A‐B): CA‐M, Blue; CA‐F, Red. Data are mean ± SEM. *P<0.05, **P<0.01, ***P<0.001, ****P<0.0001.
**Figure S3**. VM‐M3 Circulating Lipids. A Total Cholesterol (*n*=5/group), Triglycerides (*n*=4/group) and Lipase (*n*=5/group) were analyzed via colorimetry analysis. Data: EOL, Experiment 1a. Data information: Across sex differences were analyzed with unpaired t‐test. Abbreviations: SH‐M, Sham Males; CA‐M, Cancer Males; SH‐F, Sham Females; CA‐F, Cancer Females. Data are mean ± SEM. *P<0.05.
**Figure S4**. Alterations Seen in VM‐M3 Cannot Be Explained by Altered Hydration Status A Organic Phosphorous (males *n*=5/group; female *n*=4/group), Calcium (males *n*=5/group; female *n*=4/group), Sodium (*n*=3/group), Potassium (males *n*=3/group; female *n*=4/group), Chloride (males *n*=2‐3/group; female *n*=4/group), Magnesium (males *n*=3/group; female *n*=4/group) were quantified via potentiometrics. Data: EOL, Experiment 1a. Data information: Across sex differences were analyzed with unpaired t‐test. Abbreviations: SH‐M, Sham Males; CA‐M, Cancer Males; SH‐F, Sham Females; CA‐F, Cancer Females. Data are mean ± SEM. **P<0.01.
**Figure S5**. Serum and Skeletal Muscle Molecular Dynamics RT‐qPCR analysis of mRNA GPR109a in Sham Males (SH‐M) and Cancer Males (CA‐M) revealed no differences (*n*=12/group). Data: Gastrocnemius, EOL, Experiment 1a. B Total RNA was quantified via UV Spectroscopy and was significantly reduced in CA‐M compared to SH‐M (*n*=12). Data: Gastrocnemius, EOL, Experiment 1a. C BCA colorimetric assay indicated Nuclear Fraction Total Protein was significantly reduced in CA‐M (*n*=10) compared to SH‐M (*n*=12). Data: Gastrocnemius, EOL, Experiment 1a. D Immunoblotting Ubiquitination and Ponceau Staining. Data: Gastrocnemius, EOL, Experiment 1a. E Immunoblotting 20S Proteasome Core (30 kDa) and Ponceau Staining. Data: Gastrocnemius, EOL, Experiment 1a. F Immunoblotting Total NF‐κB (65 kDa) and Ponceau Staining. Data: Gastrocnemius, EOL, Experiment 1a. G Immunoblotting Cytosolic and Nuclear OGG1 confirming nuclear fractionation. Immunoblotting Nuclear Fraction Total NF‐κB (65 kDa) and FOXO3a (73kDa), and Ponceau Staining. SH‐M10 not analyzed due to tissue limitation. Data: Gastrocnemius, EOL, Experiment 1a. H Immunoblotting MuRF‐1 (43 kDa) and Ponceau Staining. Data: Gastrocnemius, EOL, Experiment 1a. I Immunoblotting Atrogin‐1 (40 kDa) and Ponceau Staining. Data: Gastrocnemius, EOL, Experiment 1a. Data information: Within group differences across time were analyzed with One‐Way ANOVA with Fischer LSD post‐hoc for ≤3 comparisons (A). Differences across groups at each timepoint were analyzed with unpaired t‐test (AD). Prior to ANOVA cytokine analysis, robust regression and outlier removal (ROUT) with coefficient Q=1% was used as non‐physiologic/error values were detected (A). Abbreviations: OGG1, 8‐Oxoguanine‐DNA Glycosylase 1. Data are mean ± SEM. ^ns^ *P*>0.05, *P<0.05, ***P<0.001.
**Figure S6**. Ketone Diester Food Incorporation Alters Systemic Metabolism and is Well Tolerated. A Baseline food intake, bodyweight, and age (*n*=12/group). Data: Experiment 4c. B Bodyweight tracked daily with step‐wise increase in KDE indicating tolerability of 30%KDE incorporated with highly palatable standard diet (HPSD) (*n*=4). Data: Experiment 4b. C Blood Glucose and Ketones were quantified via enzyme interaction for animals fed 20% (*n*=5), 25% (*n*=3), 30% (*n*=5) Ketone Diester (KDE) on top of standard diet. 30%KDE induced largest alterations in systemic metabolism. Data: Experiment 4a. Data information: Across group differences were analyzed with One‐Way ANOVA with Fischer LSD post‐hoc for ≤3 comparisons (A, C). Differences within groups (C) at each timepoint were analyzed with unpaired t‐test. Differences compared to Day 0 were compared with One‐Way ANOVA with Dunnett post hoc (B). Colors (C): Post‐KDE, Green. Data are mean ± SEM. *P<0.05, **P<0.01, ***P<0.001, ****P<0.0001.
**Figure S7**. Bodyweights Were Controlled Prior to LPS Experimentation. A Baseline bodyweight for animals prior to administration of 10mg/kg lipopolysaccharide with either 4mL water (LPS, *n*=4) or 4mL/kg KDE (KDE+LPS, *n*=5) gavage. Data: Experiment 5b. B Baseline bodyweight for animals prior to administration of 10mg/kg lipopolysaccharide with LPS (*n*=4) or KDE+LPS (*n*=4) gavage and pair‐feeding. Data: Experiment 5c. Data information: Difference across groups were analyzed with unpaired t‐test for individual comparisons (A‐B). Data are mean ± SEM. ^ns^ *P*>0.05.
