# Supplementary material for: Ketone Bodies Attenuate Wasting in Models of Atrophy
Source: J Cachexia Sarcopenia Muscle. 2020 Apr 2;11(4):973–96. doi: 10.1002/jcsm.12554 (PMC7432582; doi:10.1002/jcsm.12554)
Supplement: Supplementary file 1 — Data S1. Supporting information [file JCSM-11-973-s001.docx]

**Supplemental Information**

**Figure Legends (*n*-value loss):**

- Figure 1G-H; Figure 3B: Primary tumor weight not reliably isolated for *n*=1 CA-M and *n*=2 CA-F.
- Figure 1H; Figure 3B: Primary tumor and ascites fluid bioluminescence not quantified at 15min post-luciferin for *n*=1 CA-F and *n*=1 CA-M, *n*=1 CA-F in accurately isolated tissues due to logistic limitations, respectively.
- Figure 2E: Quadricep was not reliably isolated from upper thigh tissue for *n*=5 SH-M and CA-M, *n*=2 SH-F and CA-F.
- Figure 3B: Merged CA-M and CA-F weight change to bioluminescence ratio.
- Figure 3C; Figure 4B: Sample contamination and/or volume inhibited full cohort white blood cell impedance and clinical chemistry colorimetry analysis.
- Figure 5C&I; Figure S5C: Limited tissue quantity for *n*=1 SH-M (Fig. 5C & I), *n*=2 CA-M (Fig. S5C).
- Figure 6D; Figure 6G-I: Ascites fluid and tissue weight was not reliably isolated for *n*=1 KDE+VM-M3 and VM-M3 (Figure D), *n*=2 KDE+VM-M3 and VM-M3, and *n*=1 Sham (Figure 6G-I), respectively.
- Figure 6F: Insufficient serum volume for *n*=1 VM-M3.
- Figure S3; Figure S4: Sample contamination and/or volume inhibited clinical chemistry colorimetry analysis for *n*=7-10.
