# Supplementary material for: Ketone Bodies Attenuate Wasting in Models of Atrophy
Source: J Cachexia Sarcopenia Muscle. 2020 Apr 2;11(4):973–96. doi: 10.1002/jcsm.12554 (PMC7432582; doi:10.1002/jcsm.12554)
Supplement: Supplementary file 2 — Table S1. Experimental Design Experimental Design reference table for main experiments. Data information: Abbreviations: R βHB, R β‐Hydroxybutyrate; SH‐M, Sham Males; CA‐M, Cancer Males; SH‐F, Sham Females; CA‐F, Cancer Females; EOL, End of Life; VM‐M3, VM‐M3 Mouse Model of Systemic Metastasis; KDE, Ketone Diester; LPS, Lipopolysaccharide/Endotoxin. *= One unexplained animal death immediately postinoculation. Table S2. VM‐M3 Subcutaneous Versus Intraperitoneal Implantation. Overlapping phenotype observed with 1×10^6^ VM‐M3 subcutaneous and intraperitoneal implantation. Data information: Abbreviations: CA‐M, Cancer Males; SH‐M, Sham Males. Red line indicates region of interest (ROI). Color Scaling: Radiance (photons/sec/cm2/sr). Table S3. Ketone Diester Physical Properties Physical properties reference table for R/S 1,3‐Butanediol Acetoacetate Diester. Data information: Abbreviations: GC/MS, Gas Chromatography Mass Spectrum Analysis. Table S4. Ketone Diester Preparation and Analysis. Preparation and confirmation analysis for R/S 1,3‐Butanediol Acetoacetate Diester chemical synthesis. Data information: Abbreviations: GRAS, Food and Drug Administration Generally Recognized as Safe; GC‐FID, Gas Chromatography Flame‐Ionization Detection. Figure S1. Baseline Sex, Bodyweight, and Age Controlled with Similar Survival in Males and Females. A Sham Males (SH‐M), Cancer Males (CA‐M), Sham Females (SH‐F), Cancer Females (CA‐F) bodyweight (SH‐M, n=20; CA‐M, n=20; SH‐F, n=18; CA‐F, n=18), age (SH‐M, n=20; CA‐M, n=20; SH‐F, n=18; CA‐F, n=18), and daily food intake (SH‐M, n=12; CA‐M, n=12; SH‐F, n=11; CA‐F, n=12) were matched at baseline. Data: Bodyweight and Age, Experiment 1a&b; Food Intake, Experiment 1a. B Sham Males (SH‐M) week 1 (n=4), SH‐M week 2 (n=4), SH‐M week 3 (n=5), Cancer Males (CA‐M) week 1 (n=4), CA‐M week 2 (n=4), CA‐M week 3 (n=5), SH‐F week 1 (n=5), SH‐F week 2 (n=5), and SH‐F week 3 (n=6), Cancer Females (CA‐F) week 1 (n=5), CA‐F week 2 (n=5), CA‐F week 3 (n=6) wer [file JCSM-11-973-s002.pptx]

## Slide 1
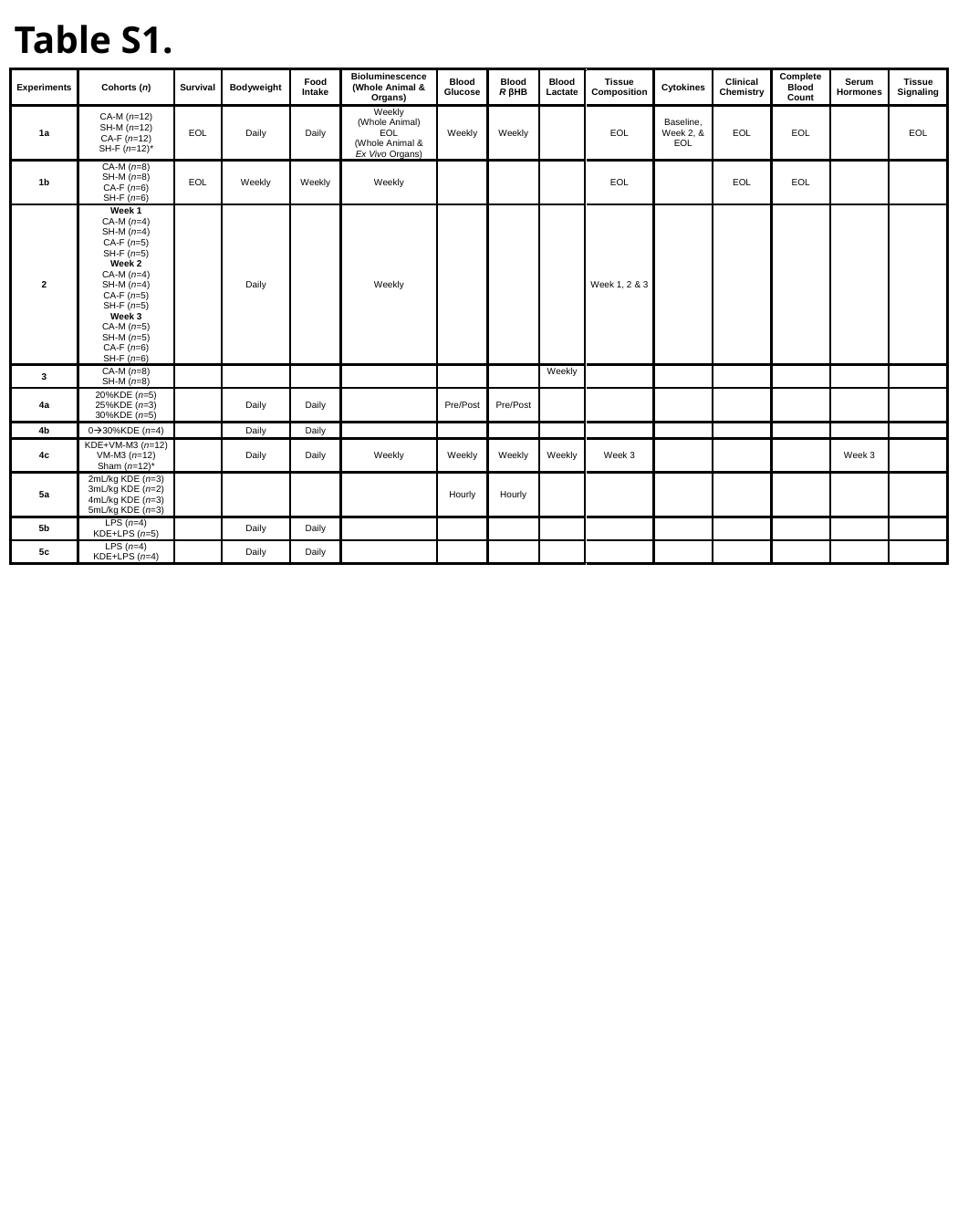

Table S1.

## Slide 2
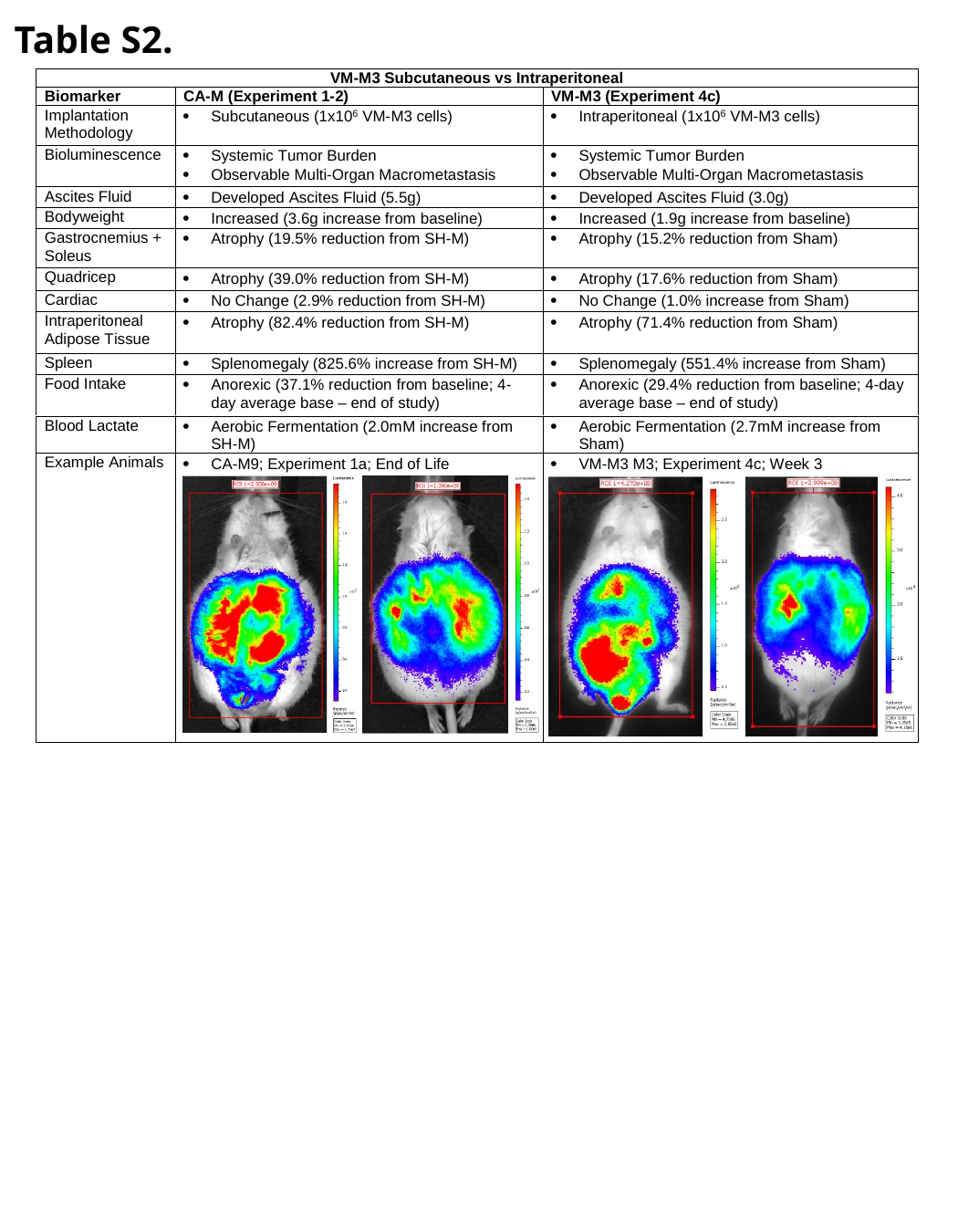

Table S2.

## Slide 3
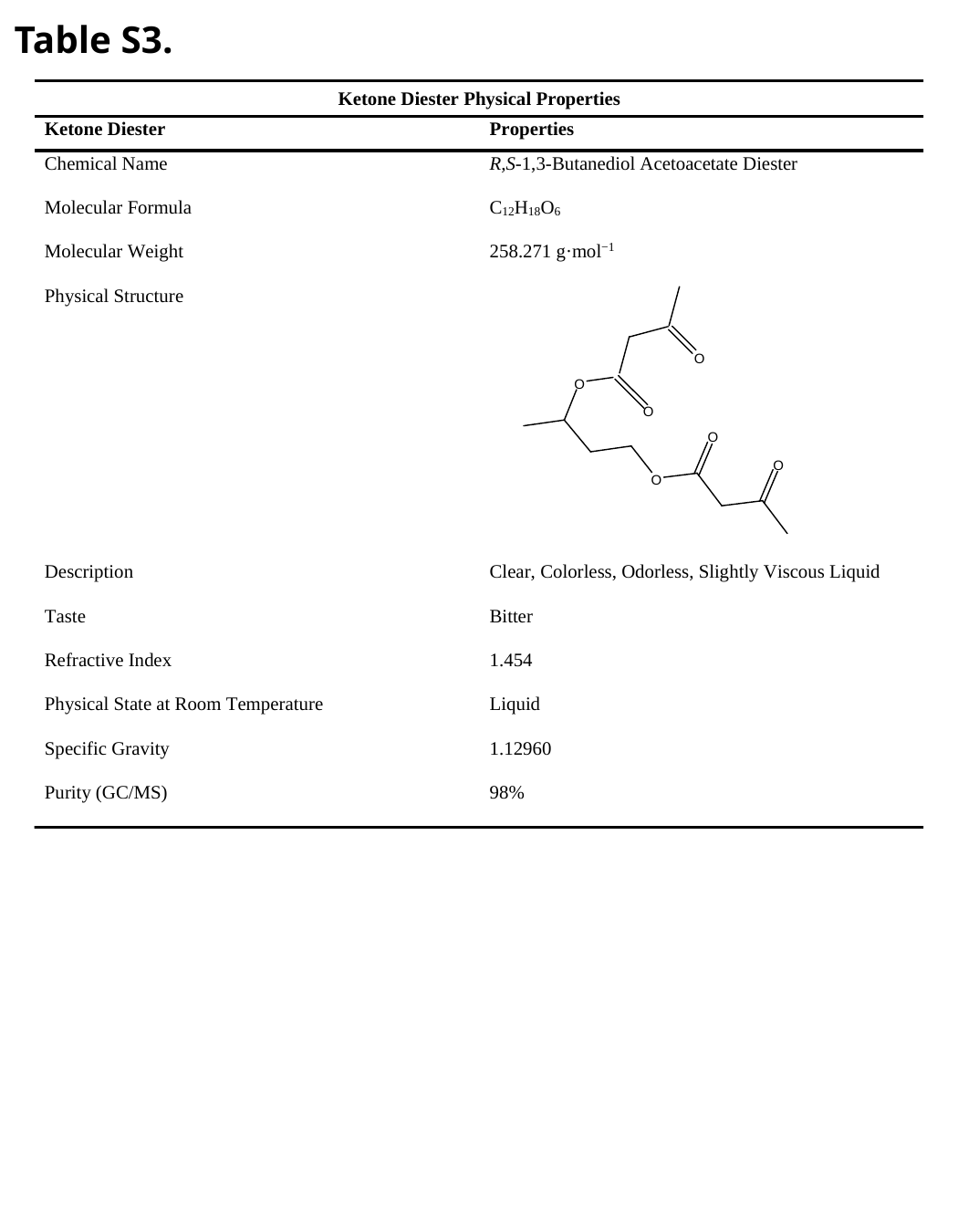

Table S3.

## Slide 4
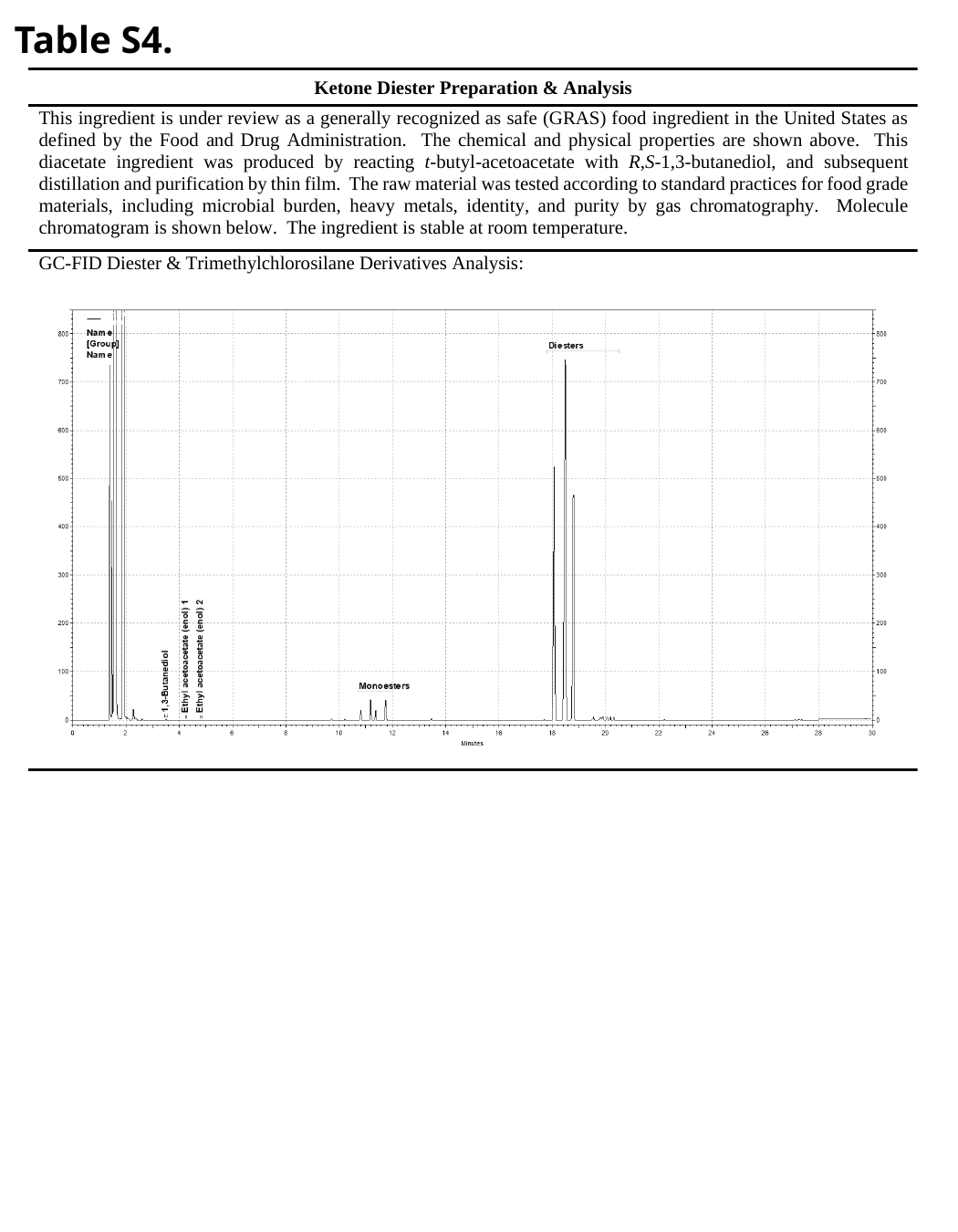

Table S4.

## Slide 5
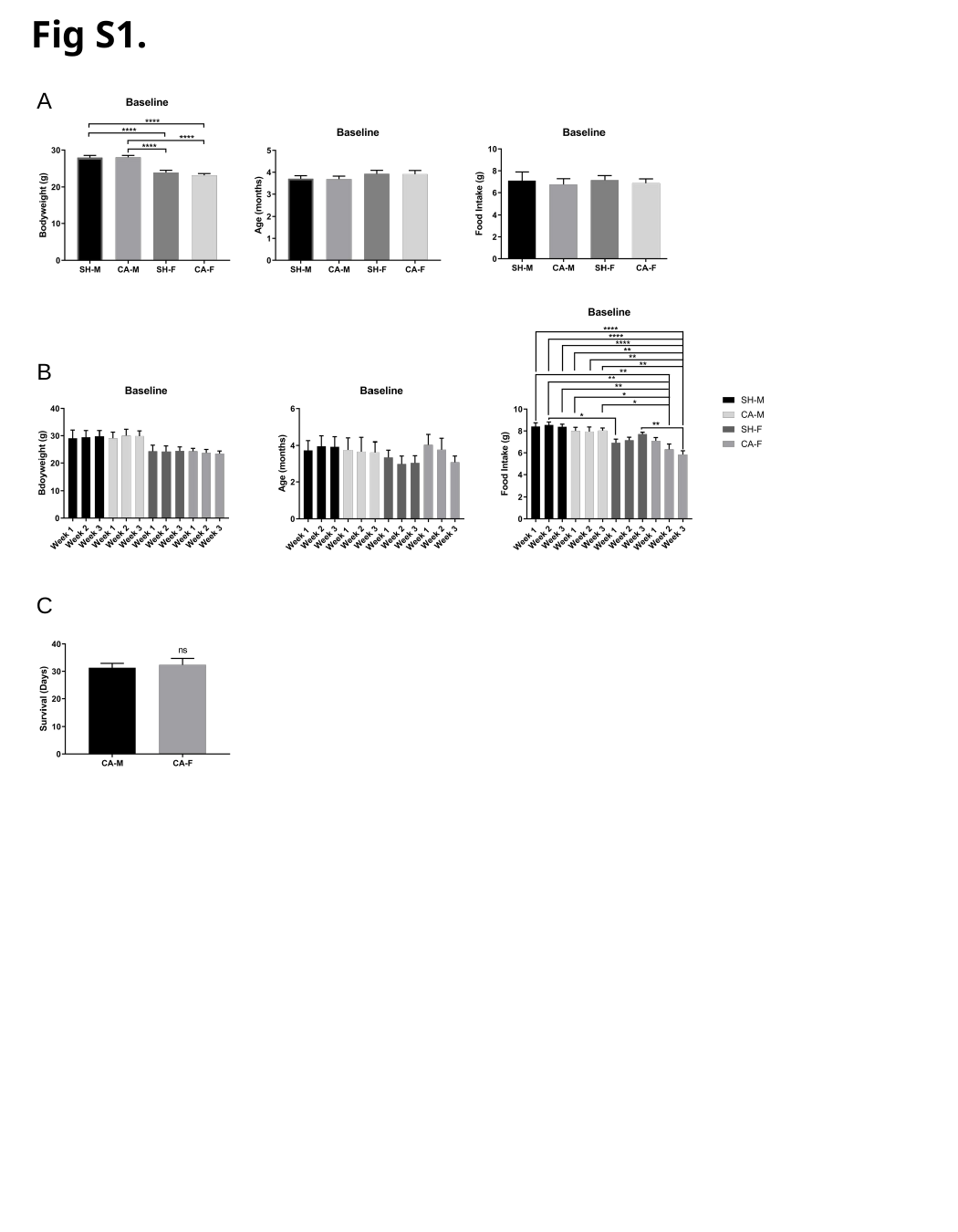

Fig S1.
A
B
C

## Slide 6
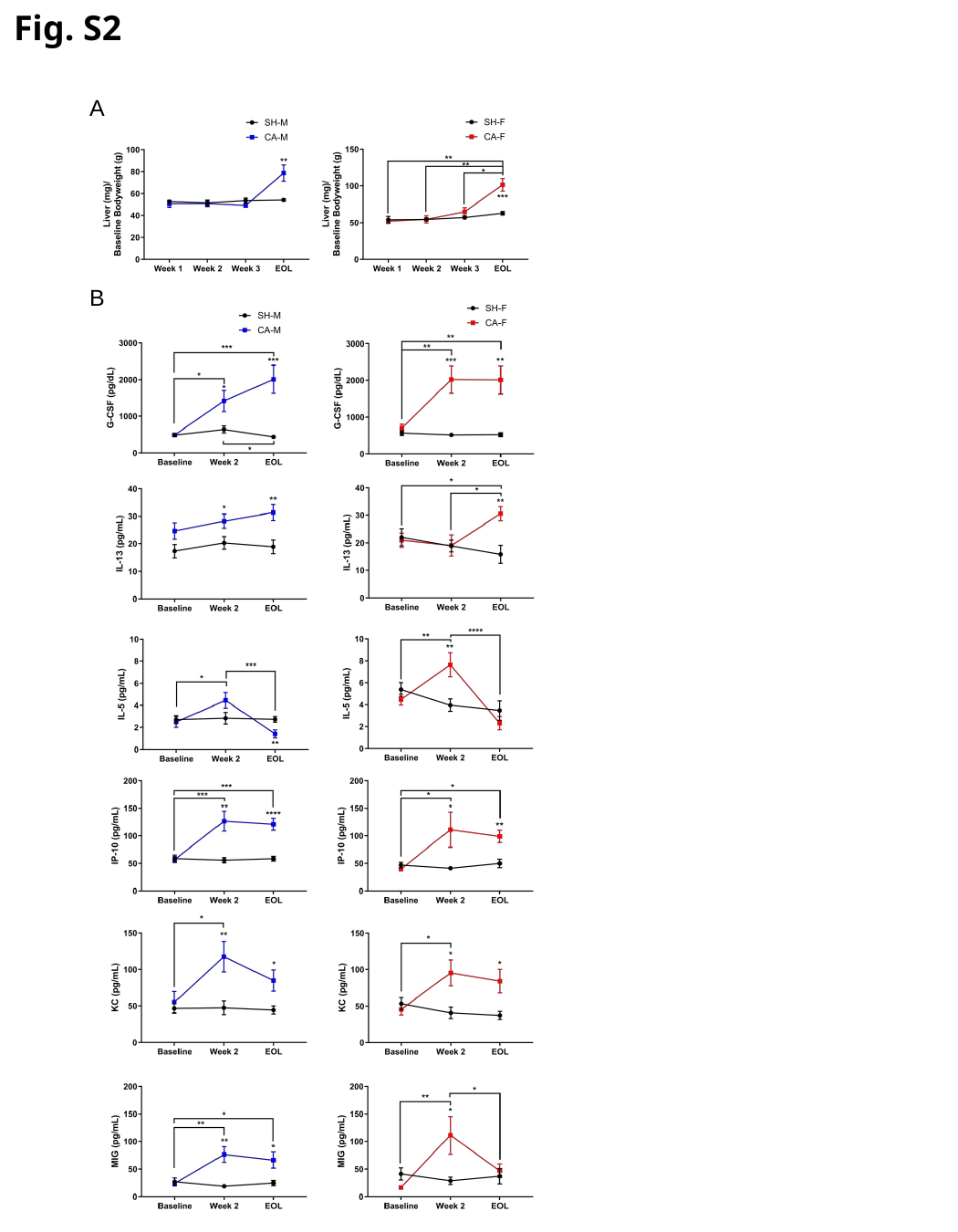

Fig. S2
A
B

## Slide 7
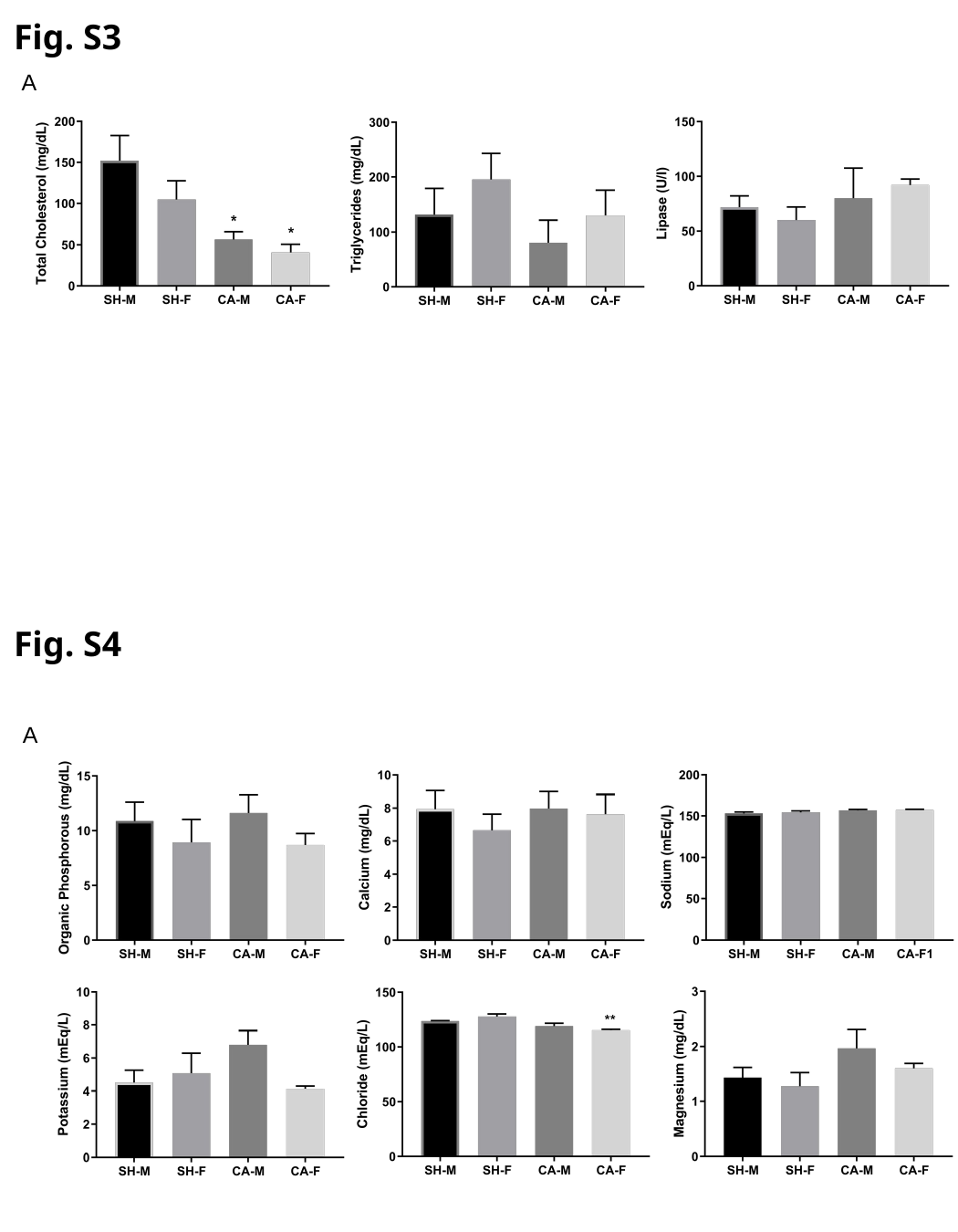

# Fig. S3
A
Fig. S4
A

## Slide 8
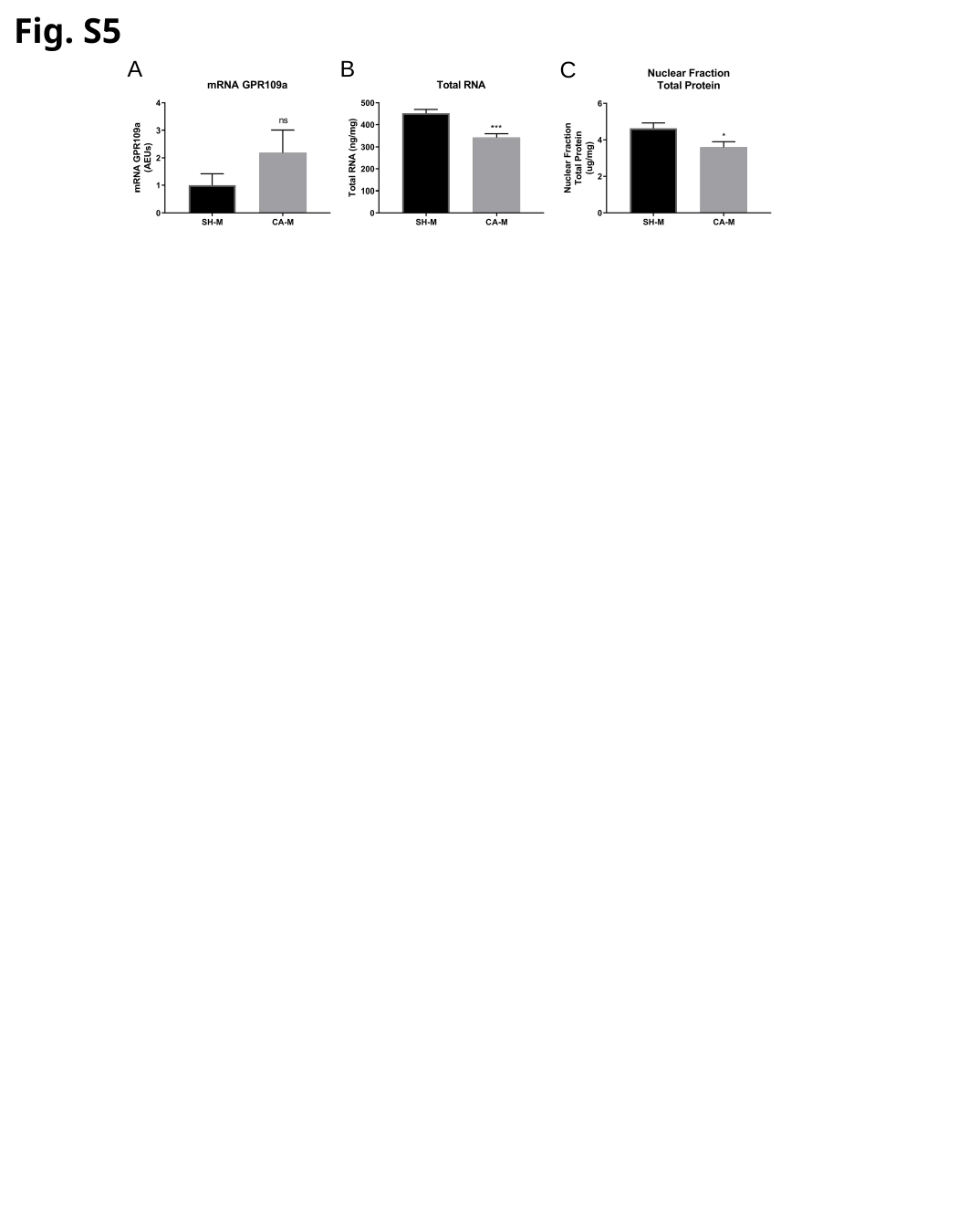

# Fig. S5
A
B
C

## Slide 9
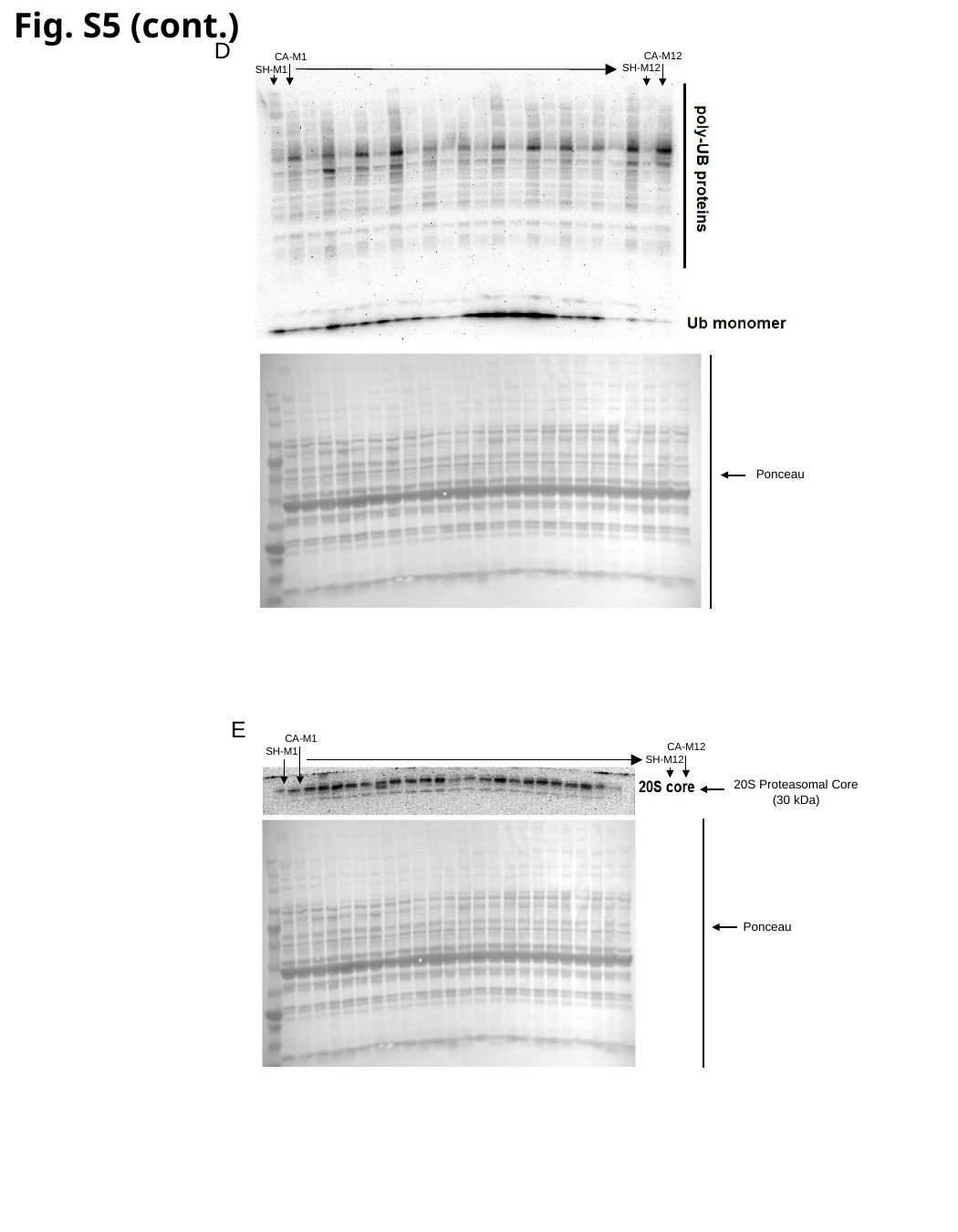

Fig. S5 (cont.)
D
CA-M12
CA-M1
SH-M12
SH-M1
Ponceau
E
CA-M1
CA-M12
SH-M1
SH-M12
20S Proteasomal Core (30 kDa)
Ponceau

## Slide 10
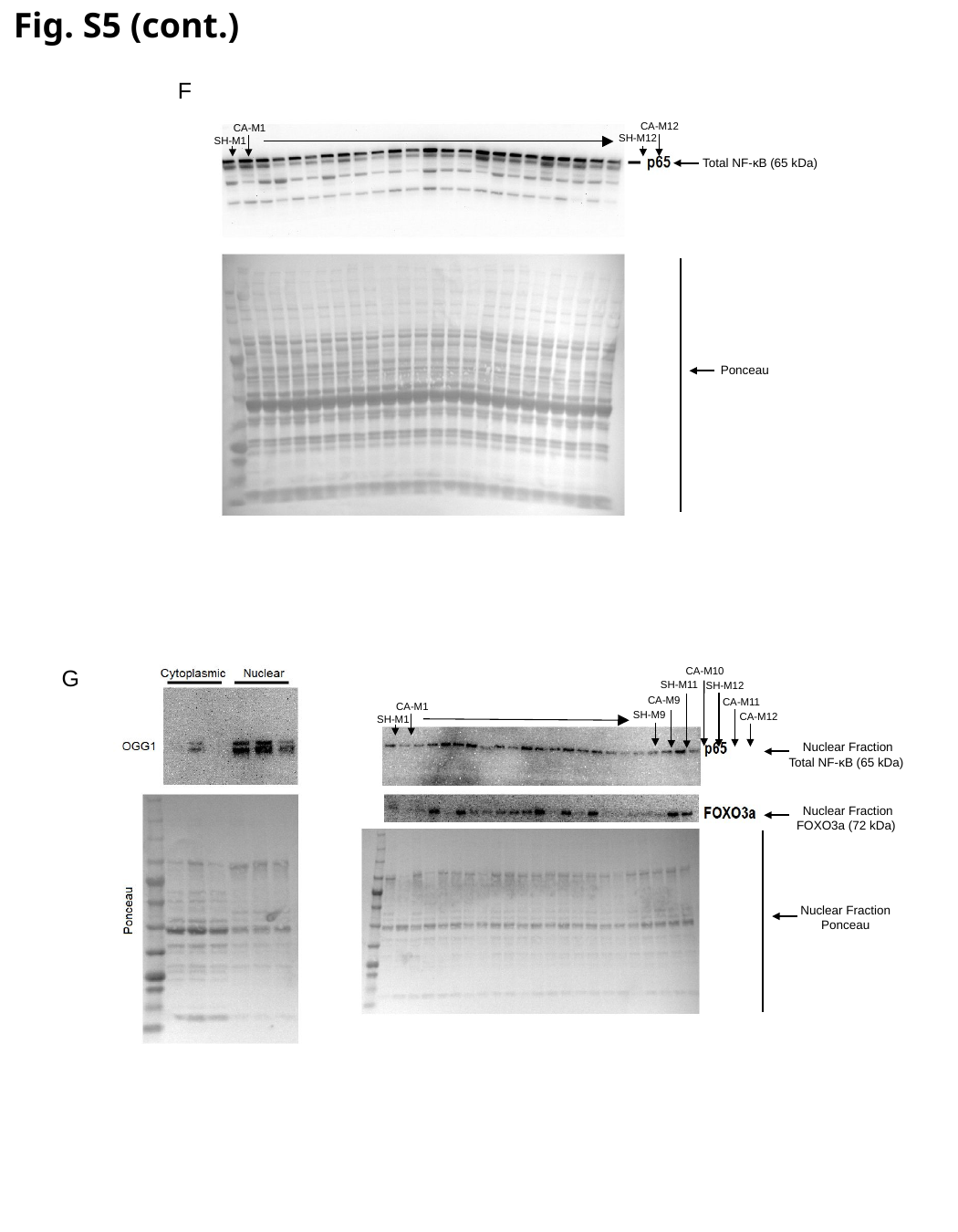

Fig. S5 (cont.)
F
CA-M12
CA-M1
SH-M12
SH-M1
Total NF-κB (65 kDa)
Ponceau
G
CA-M10
SH-M11
SH-M12
CA-M9
CA-M11
CA-M1
SH-M9
CA-M12
SH-M1
 Nuclear Fraction
Total NF-κB (65 kDa)
 Nuclear Fraction
FOXO3a (72 kDa)
Nuclear Fraction
Ponceau

## Slide 11
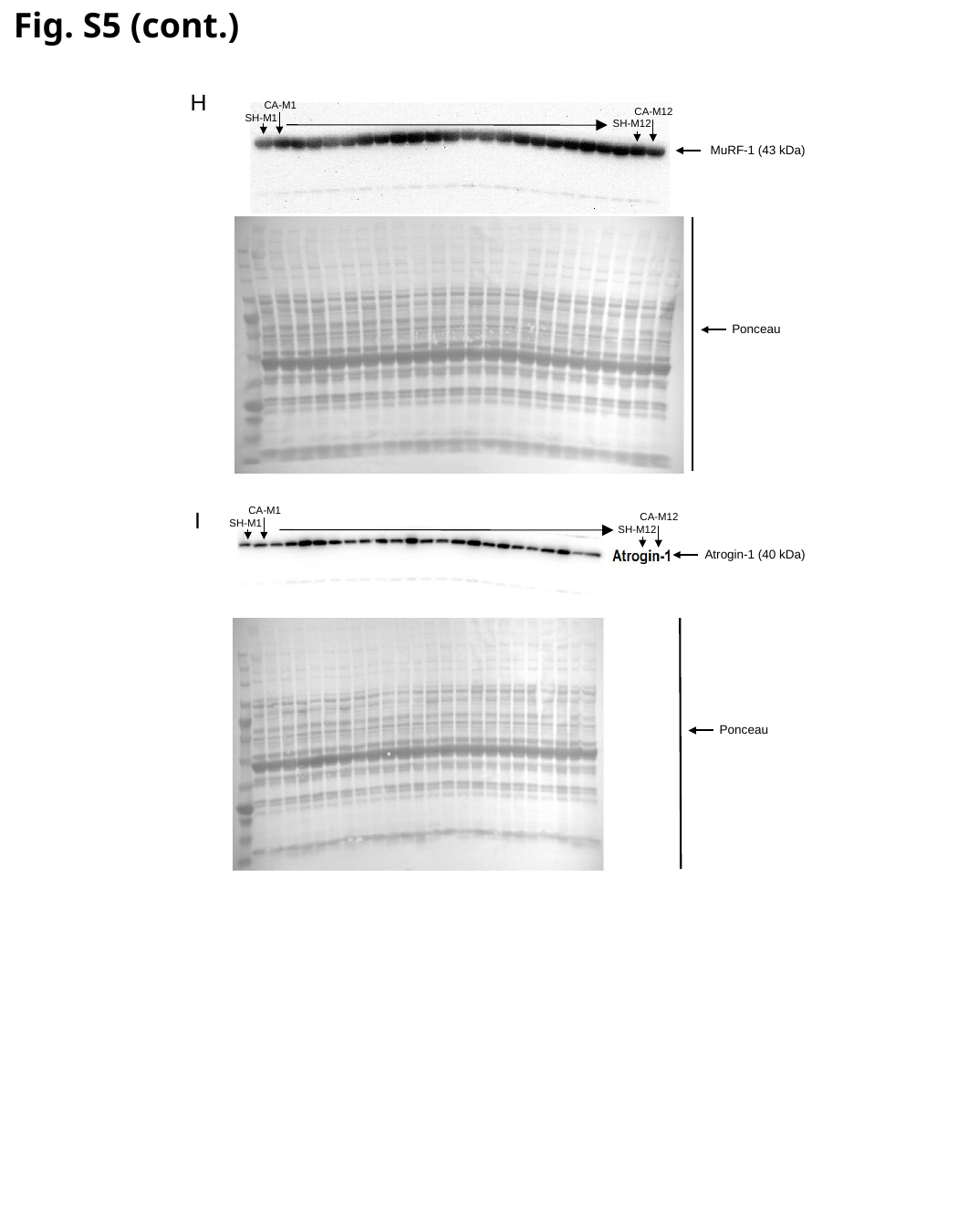

Fig. S5 (cont.)
H
CA-M1
CA-M12
SH-M1
SH-M12
MuRF-1 (43 kDa)
Ponceau
CA-M1
I
CA-M12
SH-M1
SH-M12
Atrogin-1 (40 kDa)
Ponceau

## Slide 12
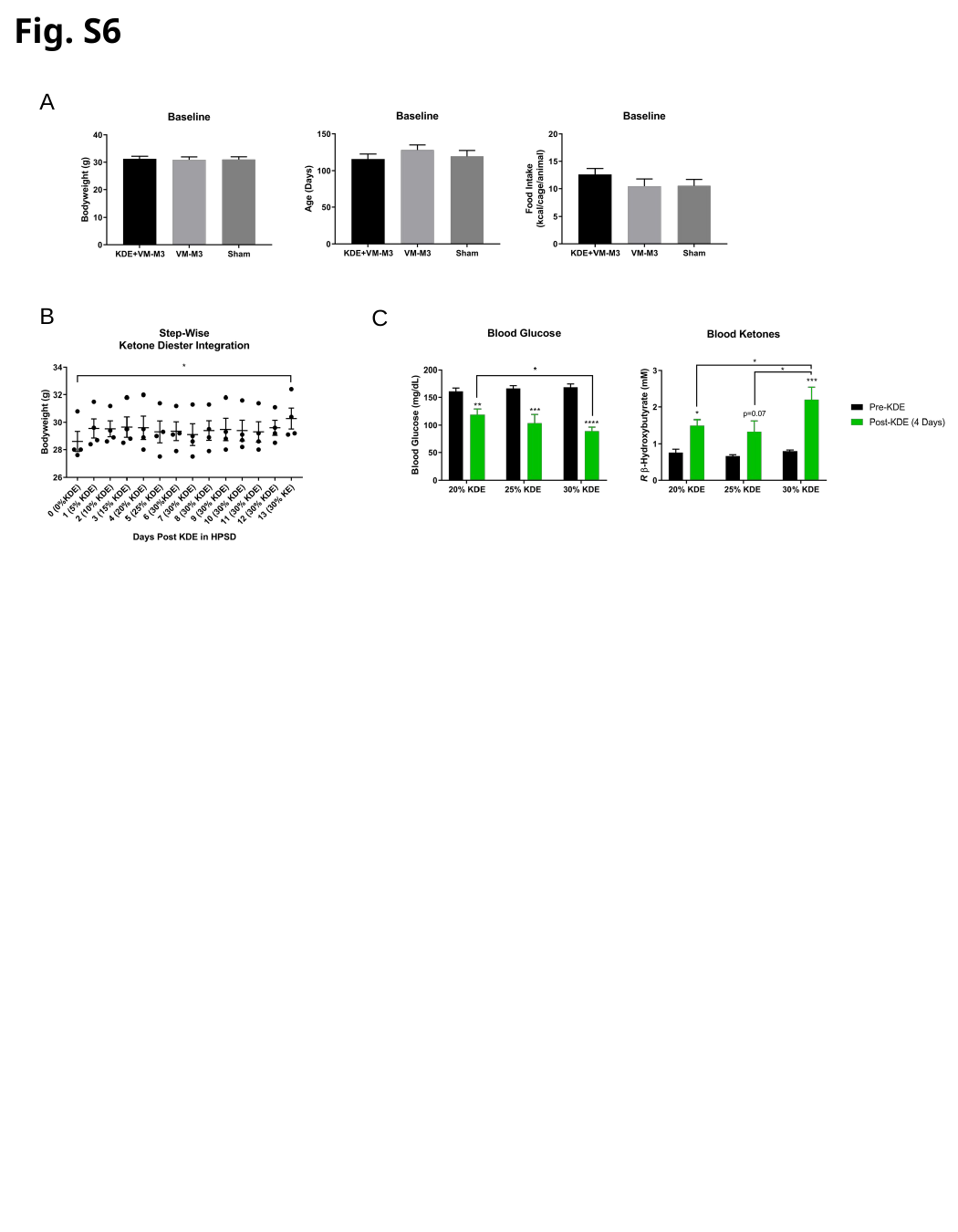

# Fig. S6
A
B
C

## Slide 13
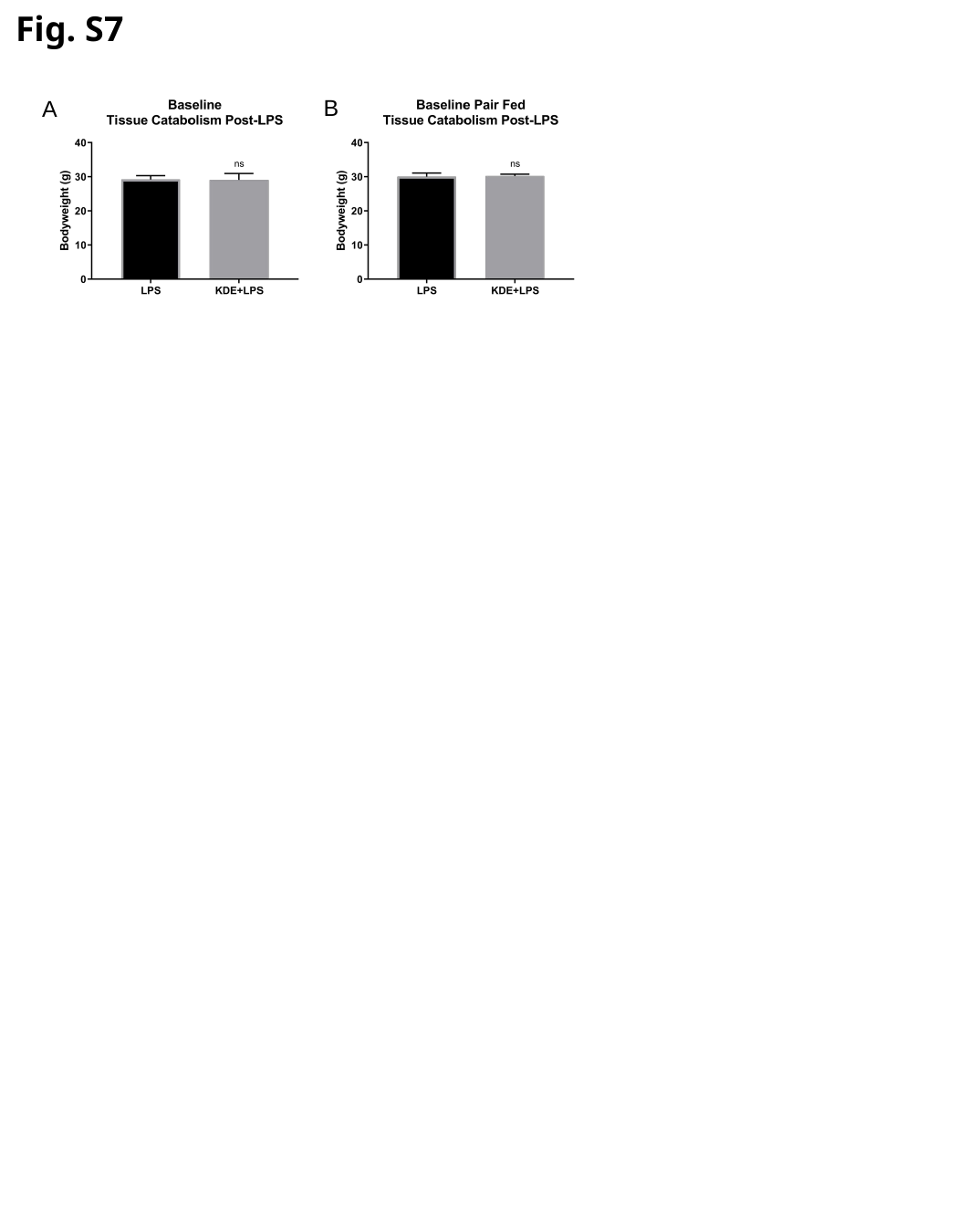

# Fig. S7
B
A
